# Fluorescent Analysis of K_M_ and V_max_ Values for Methyl-Dependent Restriction Endonucleases

**DOI:** 10.3390/biom16081217

**Published:** 2026-08-20

**Authors:** Vladislava Martyshova, Sergey Sedykh

**Affiliations:** 1 Faculty of Natural Sciences, School of Advanced Engineering Studies, Novosibirsk State University, Lyapunova St., 1, 630090 Novosibirsk, Russia; v.martyushova@g.nsu.ru; 2 Institute of Chemical Biology and Fundamental Medicine, Siberian Branch of Russian Academy of Sciences, Lavrentieva Ave., 8, 630090 Novosibirsk, Russia

**Keywords:** restriction endonucleases, DNA methylation, FRET, enzyme kinetics, Michaelis–Menten constant, reaction rate

## Abstract

Methyl-dependent restriction endonucleases are promising tools for analyzing eukaryotic DNA methylation patterns. However, quantitative assessment of their substrate specificity requires the determination of the kinetic parameters of enzymatic reactions. Here, we present a method for determining initial reaction rates based on fluorescent probes and real-time monitoring of changes in fluorescence intensity. Initial rates of methyl-dependent *Gla*I and *Bls*I restriction endonucleases were determined as the slope of the linear part of the kinetic curves, after which the Michaelis–Menten constants (K_M_) and reaction rates (V_max_) were calculated using nonlinear regression. For both enzymes, K_M_ values were determined for the first time, indicating a high affinity of the methyl-dependent restriction endonucleases for methylated sites. K_M_ values for fully methylated duplexes were in the range of (4.4–7.0)·10^2^ nM for *Gla*I and 2.4–55 nM for *Bls*I. K_M_ values were significantly lower for the hemimethylated duplexes: (1.9–8.0)·10^2^ nM for *Gla*I and (0.7–15.0)·10^2^ nM for *Bls*I; and even lower for unmethylated duplexes: (27–46)·10^2^ nM for *Gla*I and (0.28–23)·10^2^ nM for *Bls*I. Maximum reaction rates varied within relatively narrow limits: V_max_ values were in range (4.5–19.5)·10^−4^ nM/s for *Gla*I and (1.4–18)·10^−4^ nM/s for *Bls*I, respectively. V_max_ values were depended weakly on the degree of methylation compared to the K_M_. The proposed fluorescence method was applied to determine the kinetic parameters of methyl-dependent restriction endonucleases for the first time. It may serve as a simpler and more environmentally friendly alternative to traditional electrophoretic approaches that use radioactive labels.

## 1. Introduction

DNA methylation is a key epigenetic mechanism regulating gene expression, playing a fundamental role in the adaptation of organisms to changing environmental conditions [1]. In plants, changes in methylation profiles in response to stress factors such as drought, soil salinity, or pathogen exposure can activate resistance genes and form heritable epigenetic memory, which opens up prospects for breeding resistant varieties under climate change [2]. In the human body, DNA methylation is critically important for the development and maintenance of homeostasis, and its disturbances are associated with a wide range of diseases, including autoimmune, neurodegenerative, and oncological pathologies [3,4]. Analysis of specific methylation patterns is necessary both for identifying markers relevant for the diagnosis, prognosis, and personalized therapy of human diseases [5], and for the development of agricultural technologies aimed at increasing plant resistance, which overall makes it a vital tool in biomedicine and agriculture [6,7].

DNA methylation typically occurs in the context of CpG-dinucleotides, where cytosine is adjacent to guanine. Under normal conditions, most CpG-islands (regions with a high CpG density) in gene promoters remain unmethylated, allowing transcription to be activated. However, when CpG-islands become methylated, it can lead to the repression of the corresponding genes’ expression, similar to how methylation of gene promoters affects gene activity [8]. This process is crucial for cell differentiation, since different cells activate or repress specific genes according to their functions [9]. Additionally, methylation plays a key role in suppressing the activity of mobile genetic elements, such as transposons, which can cause genomic rearrangements and mutations. [10].

Currently, bisulfite sequencing is the primary method for determining DNA methylation [11]. However, several significant limitations are associated with this technique. It results in high degradation of the analyzed DNA due to harsh reaction conditions, which involve prolonged incubation in an acidic environment at elevated temperatures. This degradation necessitates a substantial amount of starting material and can lead to fragment loss. Furthermore, the method is critically sensitive to the completeness of conversion; incomplete transformation of unmethylated cytosines can produce false positive results [12]. To address the challenges of aggressive chemical processing in DNA sample analysis, Oxford Nanopore Technologies has developed an approach that allows for the direct detection of DNA methylation modifications, such as 5mC, 5hmC, and 6mA, during standard sequencing [13,14]. The main drawbacks of this method include its high dependence on the accuracy of bioinformatics analysis algorithms and reference databases for model training. As a result, the current accuracy of this method does not match that of bisulfite sequencing, particularly at low read coverage [15,16]. Therefore, there is a clear need for new methods that can reliably construct a comprehensive picture of the epigenetic landscape of genomes.

**Figure 1:**
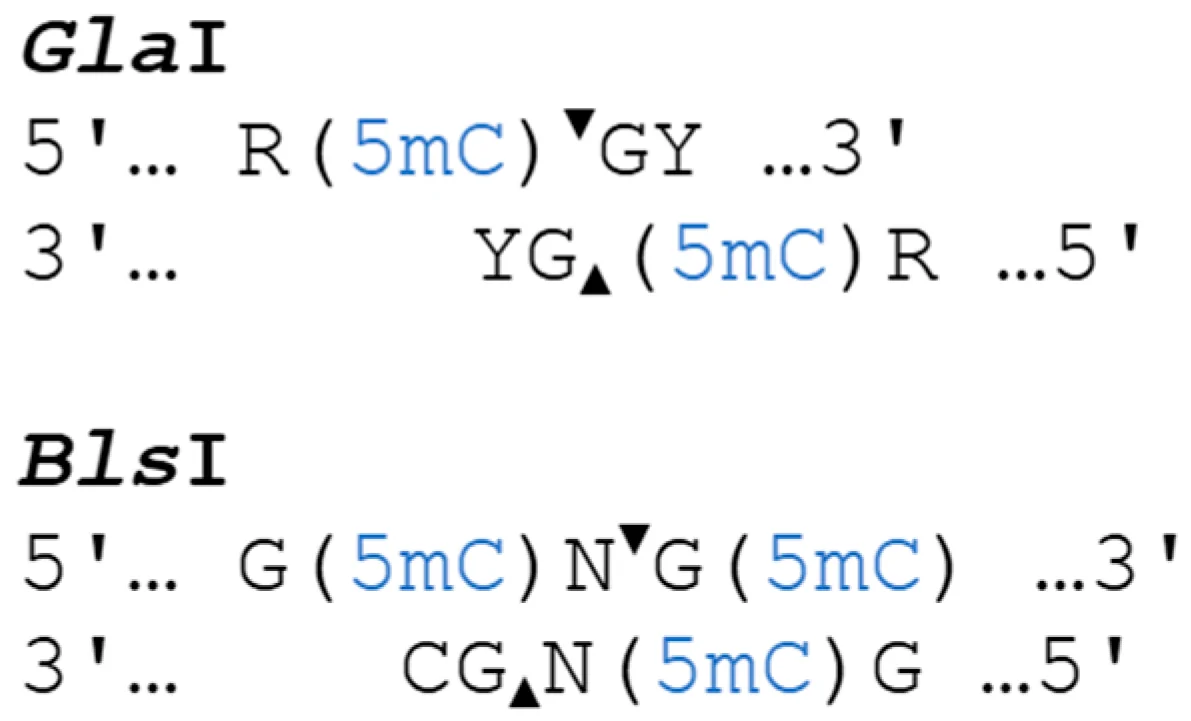
*Gla*I and *Bls*I restriction sites.

Potential tools for the efficient detection of methylation markers include specialized enzymes—methylation-dependent restriction endonucleases [17,18]. These enzymes are capable of specifically fragmenting only methylated DNA. In this study, we used methylation-dependent restriction endonucleases *Gla*I isolated from a strain of *E. coli* carrying the cloned *Gla*I gene from the *Glacial ice bacterium* Gl29, which has a recognition site R(5mC)↓GY (Figure 1), and *Bls*I isolated from the *Bacillus simplex* 23, whose recognition site is a DNA sequence containing at least two 5mC: RYN↑RY/YR↓NYR, where 5mC (5-methylcytosine) is an epigenetic marker of higher organisms, including humans [19].

Due to differences in the specific sites of the selected methylation-dependent restriction enzymes, they can be used for different purposes in the context of further constructing the epigenetic landscape of the genome. For instance, *Gla*I is suitable for searching for point methylation sites in the genome [20,21], while *Bls*I is more promising for searching for hypermethylated regions [22]. It should be noted, however, that both restriction endonucleases have degenerate recognition sites. Before utilizing these enzymes to study methylation patterns in eukaryotic genomes, it is crucial to confirm that both enzymes exhibit a high affinity for all variants of their specific methylated sites, while demonstrating low affinity for unmethylated sites. In this manuscript, we analyze their kinetic parameters related to specific and nonspecific binding behavior, using the Michaelis–Menten model.

Important characteristics of the enzymatic reactions are the Michaelis–Menten constant—K_M_ and the maximum reaction rate (velocity)—V_max_. The Michaelis–Menten constant is used to estimate the functional chemical affinity of an enzyme and substrate, which in the case of the restriction endonucleases, is determined by the number of weak interactions between the enzyme active site and corresponding oligonucleotide [23]. Determination of the kinetic parameters of methylation-dependent restriction endonucleases is important for quantitative analysis of their substrate specificity. Comparative analysis of these parameters under specific (with a fully methylated site) and nonspecific (with a hemimethylated or unmethylated site) binding conditions allows an accurate characterization of enzymatic selectivity, which directly impacts the accuracy and interpretation of data when analyzing methylation patterns in eukaryotic genomes [24]. In this work, we hypothesized that kinetic parameters depend on the methylation level of the oligonucleotide substrate, and that for methyl-dependent restriction endonucleases, K_M_ values should be higher in the case of an unmethylated oligonucleotide compared to a methylated (specific) one, indicating a lower functional affinity of the enzyme’s active site for the non-specific substrate.

This paper is devoted to a method for determining parameters of enzymatic reactions based on changes in fluorescence intensity over time. For this purpose, oligonucleotide duplexes containing fluorophore and quencher molecules were used. Between these molecules, fluorescence quenching occurs in the intact molecule via the Förster fluorescence emission (FRET) mechanism [25]. Recording fluorescence values in real time allows direct and continuous measurement of the reaction kinetics and calculation of the initial rate of the enzymatic reaction.

Methods using fluorescence [26] and FRET [27] registration have previously been used to analyze the catalytic activity of restriction endonucleases and their mechanism of action [28]. However, to the best of the authors’ knowledge, the approach to analyze kinetic parameters of methyl-dependent restriction endonucleases using fluorescent probes is being used for the first time.

## 2. Materials and Methods

### 2.1. Materials and Reagents

*Gla*I and *Bls*I restriction endonucleases and corresponding Buffers B and W were from SibEnzyme (Novosibirsk, Russia). The initial concentration of the enzymes was 50,000 and 5000 enzyme activity units in 1 mL for *Gla*I and *Bls*I, respectively. Other chemicals used were from Sigma Aldrich (St. Louis, MO, USA) and MP Biomedicals (Irvine, CA, USA). Oligonucleotides and probes were synthesized by solid-phase synthesis using the phosphoramidite method by the “DNA-synthesis LLC” (Moscow, Russia).

### 2.2. Preparation of Substrates Containing Fluorophore and Quencher

**Figure 2:**
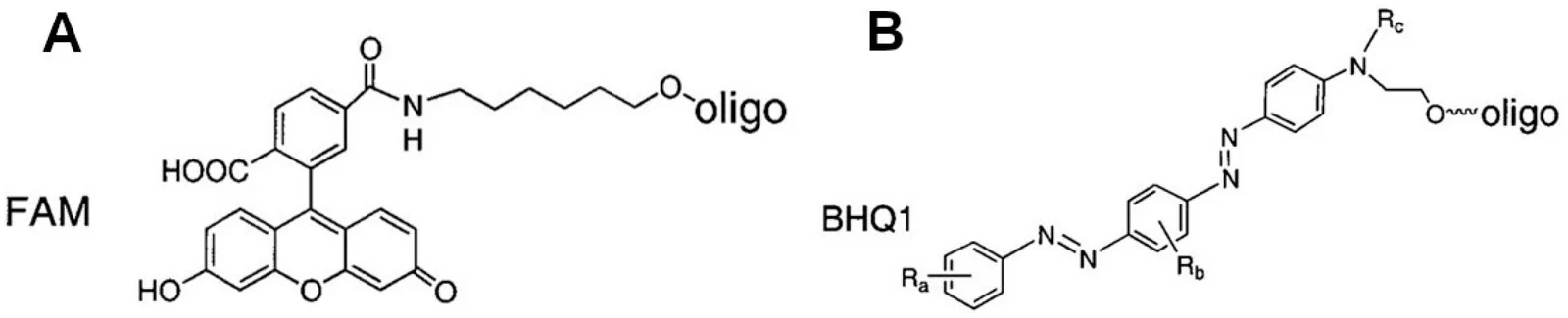
Structure of FAM (**A**) and of BHQ1 quencher (**B**) attached to the oligonucleotide.

To determine the initial reaction rate, a parameter that reflects the extent of the reaction and can be experimentally measured throughout the entire conversion should be determined. For oligonucleotides, changes in fluorescence intensity over time can serve as such a parameter [29,30]. A fluorescent label is an organic molecule with a branched conjugated π-system. By varying the substituents and the magnitude of the π-system, these molecules can have different absorption and emission ranges [31,32]. Fluorescent labels are linked to the 5′-end of the oligonucleotide via an amino linker. We used oligonucleotides that have a fluorescent label FAM at the 5′-end and a quencher BHQ1 at the 3′-end; their structures are shown in Figure 2. FAM (carboxyfluorescein) is the most common label, since the excitation maximum for its derivatives is in the range of spectral lines of argon (488 nm) and Nd:YAG (477 nm) lasers, and it also has a high quantum yield. BHQ1 (Black Hole Quencher 1) is a “dark quencher”—a substance that absorbs the excitation energy of the fluorophore and dissipates the energy as heat [31].

Table 1 shows the sequences of the oligonucleotides and probes used in this study to investigate the kinetics of *Gla*I restriction endonuclease.

Table 2 shows the sequences of the oligonucleotides used to determine the kinetics of *Bls*I restriction endonuclease.

To obtain DNA duplexes, 10 µL of each of the oligonucleotide and probe solutions (listed in Table 1 and Table 2) were mixed at an initial concentration of 100 µM; the total reaction volume was 20 µL. The resulting mixtures were heated to 60 °C in a solid-state thermostat and then slowly cooled to room temperature. The duplex concentration in the resulting stock solutions was 50 µM.

### 2.3. Analysis of Fluorescence Intensity Changes in Real Time

**Figure 3:**
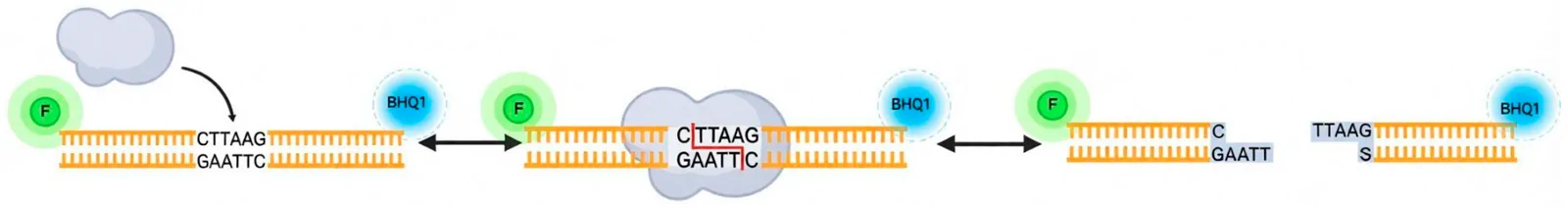
Mechanism of the enzymatic restriction reaction. Endonuclease, which recognizes a specific nucleotide sequence and causes a double-strand break, is shown in gray; fluorophore is shown in green, quencher is shown in blue.

In our method for determining kinetic parameters, the initial rates of enzymatic reactions are determined as the slope of the linear section of plot corresponding to the fluorescence intensity versus time. Fluorescence intensity changes in our experiments occurred as a result of a methyl-directed restriction by endonuclease introducing a double-strand break at a specific site within the duplex. As a result of this break, two separate fragments occur in which fluorophore and quencher are separated from each other: fluorescence intensity increases proportionally to the accumulation of the enzymatic reaction product. The scheme of general experimental setup is shown in Figure 3.

To record fluorescence changes in the wells of a white opaque 96-well plate (Eppendorf, Hamburg, Germany), a total volume of 100 µL of the reaction mixture was combined with varying amounts of duplex added to each well. To make a concentration gradient, three solutions with different concentrations were prepared by dilution of a 50 µM duplex stock solution. Concentrations and volumes of the solutions are presented in Table S1 (see Supplementary Materials). Each measurement was performed in at least three replicates. If the three obtained values after averaging showed as significant standard deviation (SD) values, additional experiments were performed (up to 6–8 repetitions of one experimental point) prior to the data processing and analysis by nonlinear least-squares regression.

Reagents, except the *Gla*I enzyme, were mixed in the wells. *Gla*I was added at the very end, and the time between adding the enzyme to the first well and starting the fluorescence detection was recorded. The composition of the reaction mixtures and the initial duplex concentrations are presented in Table S2 (see Supplementary Materials). As for *Gla*I, the reaction mixtures for the *Bls*I restriction endonuclease assay were mixed similarly, except the reaction mixture did not contain DMSO. The enzyme was also added at the very end, and the time between adding it to the first well and starting the fluorescence detection was recorded. The composition of the reaction mixtures and initial duplex concentrations are shown in Table S3 (see Supplementary Materials).

The 96-well plates containing the reaction mixture with *Gla*I or *Bls*I added were placed in a CLARIOstar^®^ Plus fluorometer (BMG Labtech, Ortenberg, Germany), where the reaction was thermostated at 30 °C for 40 min. Fluorescence readings were taken every 40 s; sixty fluorescence intensity readings were recorded for each well. Results were analyzed in the MARS Data Analysis V5.02 (CLARIOstar, Ortenberg, Germany). Plots of the linear dependence of the initial velocity on the substrate concentration are presented in Supplementary Data, see Figure S1.

### 2.4. Processing of Experimental Results

Experimentally obtained fluorescence intensity values for each cycle were used to obtain initial rates for each substrate. Changes in emission intensity, and consequently the quantum yield, were directly proportional to the accumulation of the reaction product. Quantum yield was determined by normalizing the emission intensities to the initial intensity value. We used a continuous kinetics recording method based on the normalization of the fluorescence signal to substrate reference points, which is standard and has been used in other studies [26]. For each molar concentration of the duplex tested, we recorded the donor emission intensity before hydrolysis and after reaching the reaction plateau. This allowed us to correlate the maximum fluorescence quenching with zero conversion and the fluorescence level after reaching the plateau with 100% reaction yield. We calculated the molar rate of enzymatic catalysis V_0_ in the initial linear region by scaling the slope of the kinetic curve to the total range of intensity change corresponding to the known initial substrate concentration. Initial rates for each substrate were obtained by fitting of the linear section of each graph. In this model, the slope represents the initial rate of the enzymatic reaction.

Experimental data for both enzymatic reactions were processed by nonlinear least-squares regression using the Michaelis–Menten equation to determine the kinetic parameters (K_M_ and V_max_) using a Python 3 script written with the numpy and matplotlib.pyplot libraries. The number of points used for the regression analysis was chosen to ensure the R2 value was at least 0.8.

## 3. Results

### 3.1. Substrates for Enzymatic Reactions

Double-stranded substrates were obtained as described in Section 2.2. *Gla*I restriction endonuclease recognition site is degenerate; therefore, the duplexes obtained for studying its kinetic parameters contain different numbers of recognition sites with varying degrees of the restriction site methylation (double-stranded sequences contain two, one, or zero of 5-methylcytosines): *G8m-G7m, *G8m-G7, *G8-G7 (containing two recognition sites in a row), *G8m-G3m, *G8m-G3, *G8-G3, *G5m-G6m, *G5m-G6, *G5-G6, *G18m-G19m, *G18m-G19, *G18-G19 (containing one recognition site each). Sequences of corresponding oligonucleotides are presented in Table 1.

Since *Bls*I restriction endonuclease recognition site is more degenerate, the duplexes should therefore differ not only in the degree of methylation, but also in the composition of the recognition site: N21*-N22, N17*-N20, N17*-N22, N17*-N16, Nn17*-N16 (contain the recognition site 5′-GCNGC-3′), N4*-N8, N4*-N12, N4*-Nn12, Nn4*-Nn12 (contain the recognition site 5′-ACNGC-3′/3′-TGNCG-5′), N1*-N7, N1*-N2, N1*-Nn2, Nn1*-Nn2 (contain the recognition site 5′-GTNGC-3′/3′-CANCG-5′), N6*-N5, N6*-Nn5, Nn6*-Nn5 (contain the recognition site 5′-ATNGC-3′), N13*-N14, N13*-Nn14, Nn13*-Nn14 (contain the 5′-ACNGT-3′ recognition site). Sequences of corresponding oligonucleotides are presented in Table 2.

**Figure 4:**
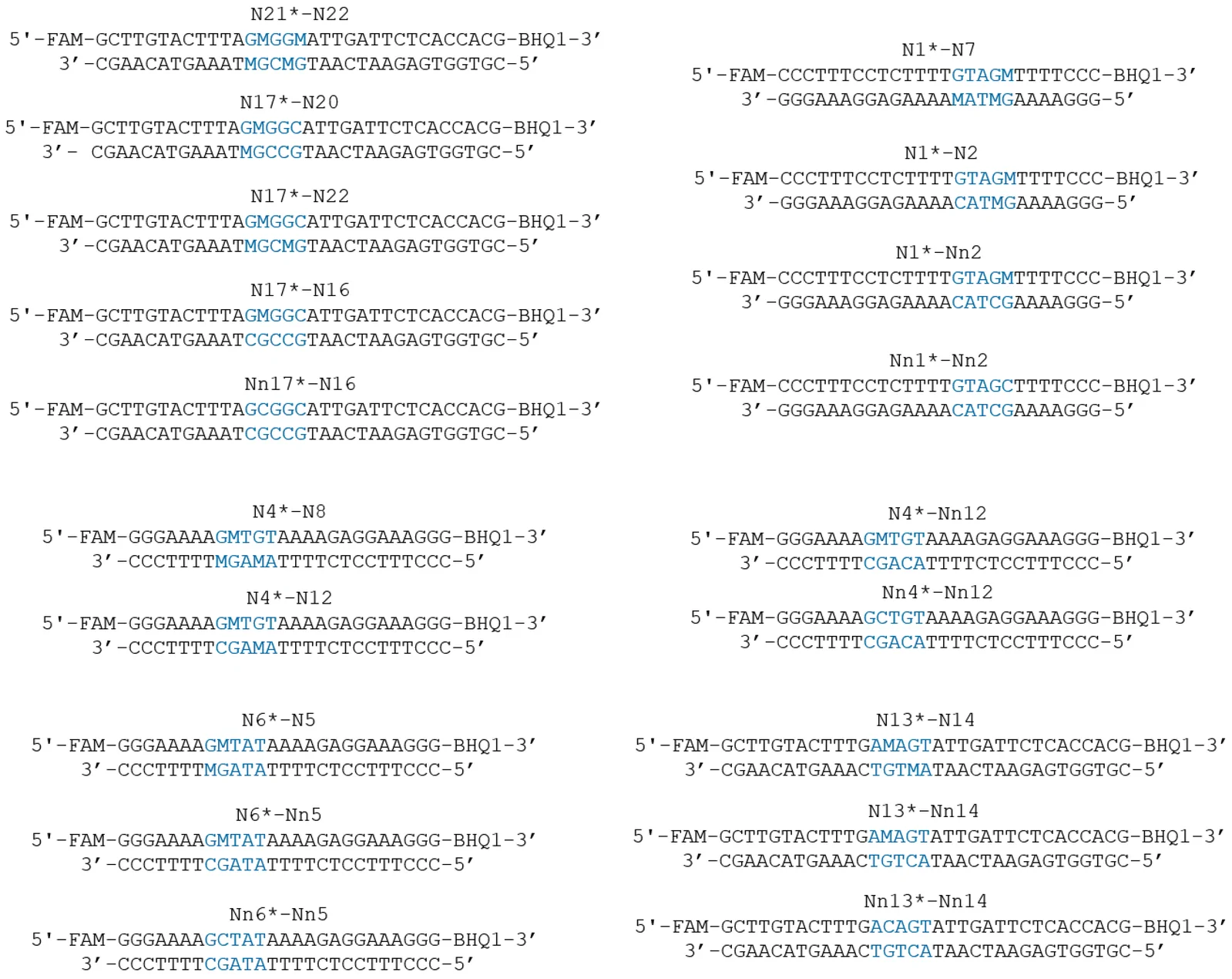
Structures of duplexes used for screening of *Bls*I kinetic parameters. Restriction sites are shown in blue. M = 5-methylcytosine; * = probes, containing 5′-FAM (carboxyfluorescein) and 3′-BHQ1.

Figure 4 shows the structures of the duplexes used to analyze the kinetic parameters of *Bls*I restriction endonuclease.

In each of the duplexes, the distance between the fluorophore and the quencher is approximately 41 Å, suggesting that quenching in this system occurs via the FRET mechanism [31].

### 3.2. Initial Rates of Enzyme Reactions

**Figure 5:**
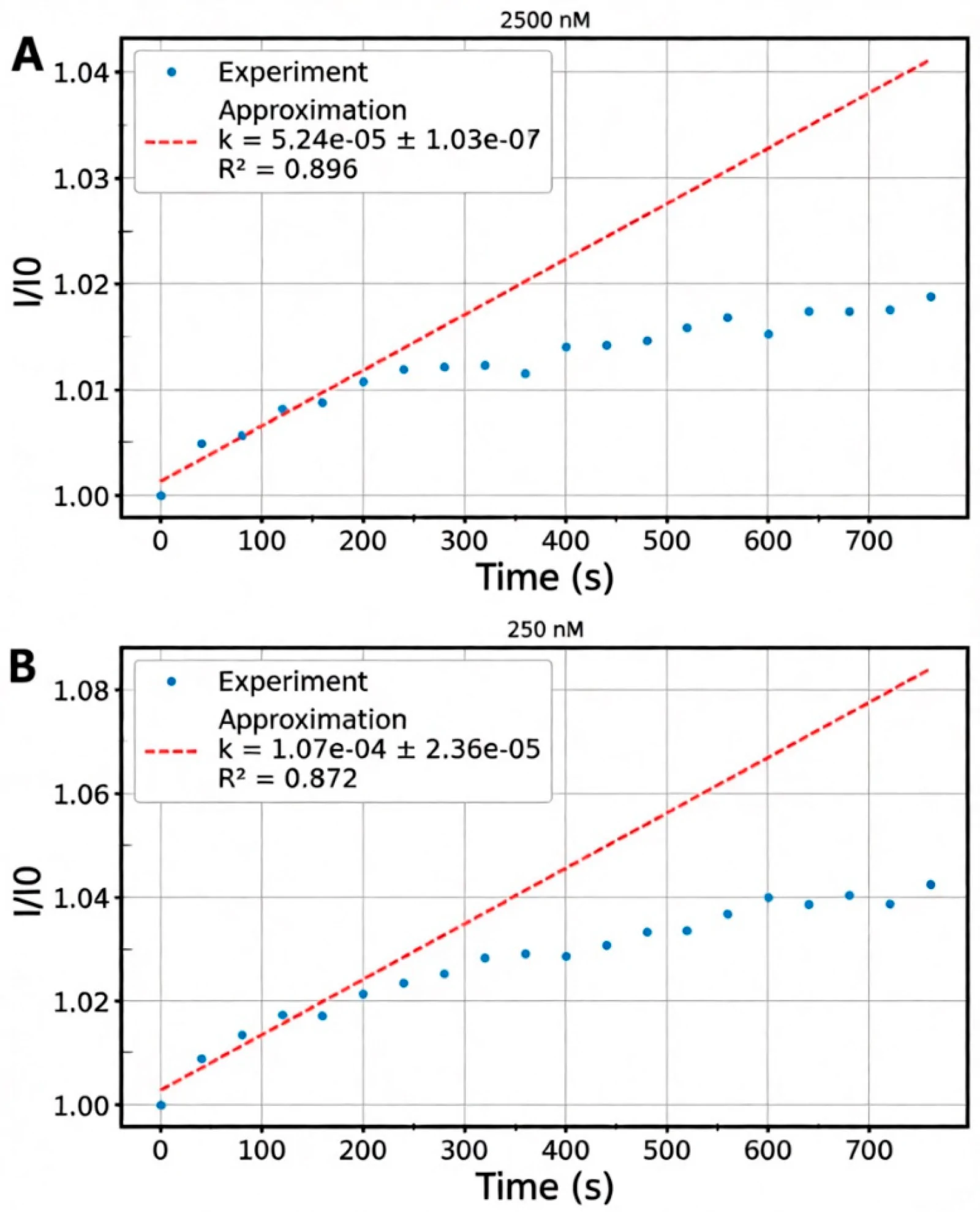
Plots corresponding to the normalized fluorescence intensity versus time for *Gla*I restriction endonuclease; concentrations of G8m-G7m duplex—2500 nM (**A**) and 250 nM (**B**)—after linearization.

By recording the fluorescence intensity over time, we obtained eight graphs for *Gm-Gn duplexes and seven graphs for Na*-Nb duplexes (where n, m, a, and b are the corresponding numbers). Each graph corresponds to a separate initial duplex concentration, which is listed above in Table 3 and Table 4 for *Gla*I and *Bls*I, respectively. Initial reaction rates for each initial duplex concentration were calculated from those graphs. As an example, Figure 5 shows graphs of the normalized fluorescence intensity (I/I_0_) versus time for the G8m-G7m duplex corresponding to concentrations of 2500 nM (Figure 5A) and 250 nM (Figure 5B). The initial rates were calculated using the first several points of each graph, with R^2^ maintained above 0.8.

### 3.3. Kinetic Parameters for Methyl-Dependent Restriction Endonucleases

Initial velocity values were processed using nonlinear least squares regression using a Python 3 script written with the numpy and matplotlib.pyplot libraries. Figure 6 shows an example of graphs from which nonlinear fitting yielded K_M_ and V_max_ values were determined for the G8m-G7m and G8-G7 duplexes. Supplementary Materials contain 31 plots corresponding to all the duplexes (see Figures S2–S32).

Using Michaelis–Menten kinetics, we obtained K_M_ and V_max_ values for *Gla*I. As shown in Table 3, K_M_ values differ for duplexes containing methylated, hemimethylated and unmethylated oligonucleotides, while V_max_ values have the same order.

As one can see from Table 3, enzyme specificity for the duplexes containing unmethylated bases (e.g., G8-G7), is lower than for methylated ones, which corresponds to the initial hypothesis.

**Figure 7:**
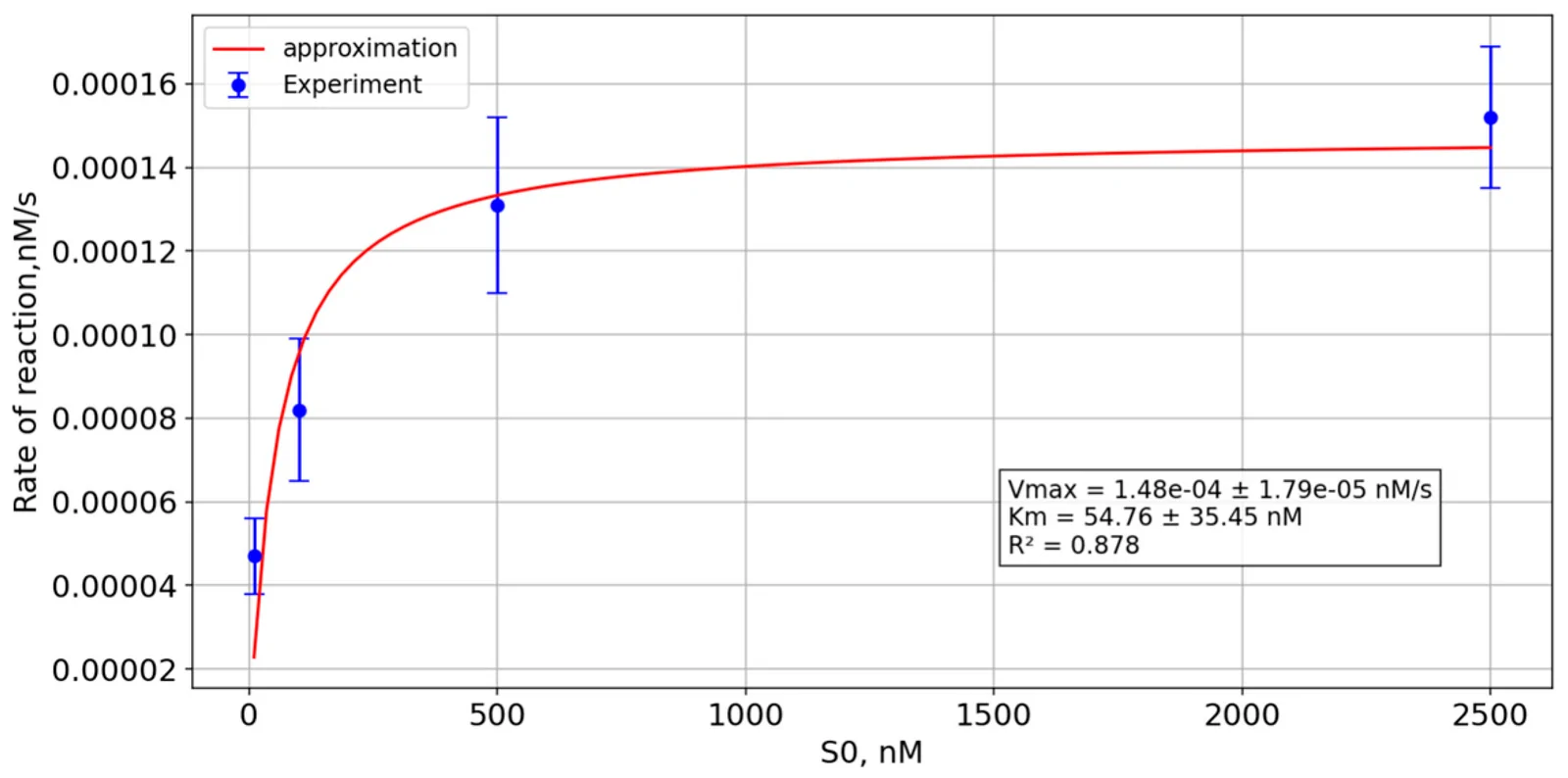
Plot corresponding to the dependence of the initial velocity on the initial substrate concentration for *Bls*I; duplexes N21*-N22; approximation by the least squares method.

**Figure 8:**
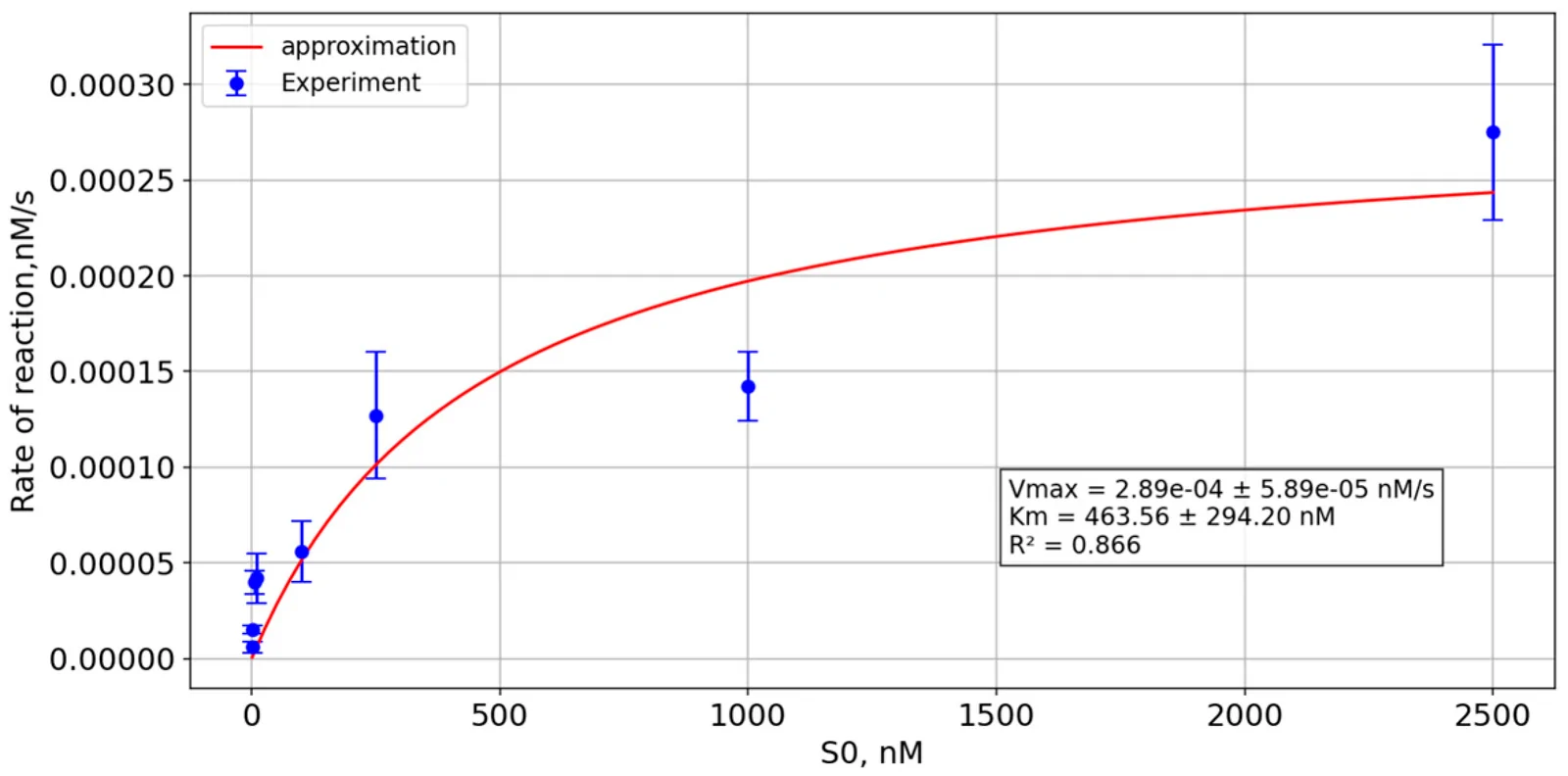
Plot corresponding to the dependence of the initial velocity on the initial substrate concentration for *Bls*I; duplexes Nn17*-N16; approximation by the least squares method.

Kinetic parameters were determined for *Bls*I using a similar approach, examples of nonlinear regression approximation graphs for the kinetic characteristics of the *Bls*I restriction endonuclease are presented in Figure 7 and Figure 8 for methylated and unmethylated oligonucleotides, respectively.

Figure 7 shows that the experimental data for the methylated duplex fit the regression curve well. Figure 8 shows the profile for the unmethylated substrate.

Table 4 presents K_M_ and V_max_ values determined for *Bls*I.

As can be seen from Table 4, the K_M_ values for the *Bls*I restriction endonuclease are lower for methylated than for semi- or unmethylated substrates, which confirms the initial hypothesis.

## 4. Discussion

**Figure 9:**
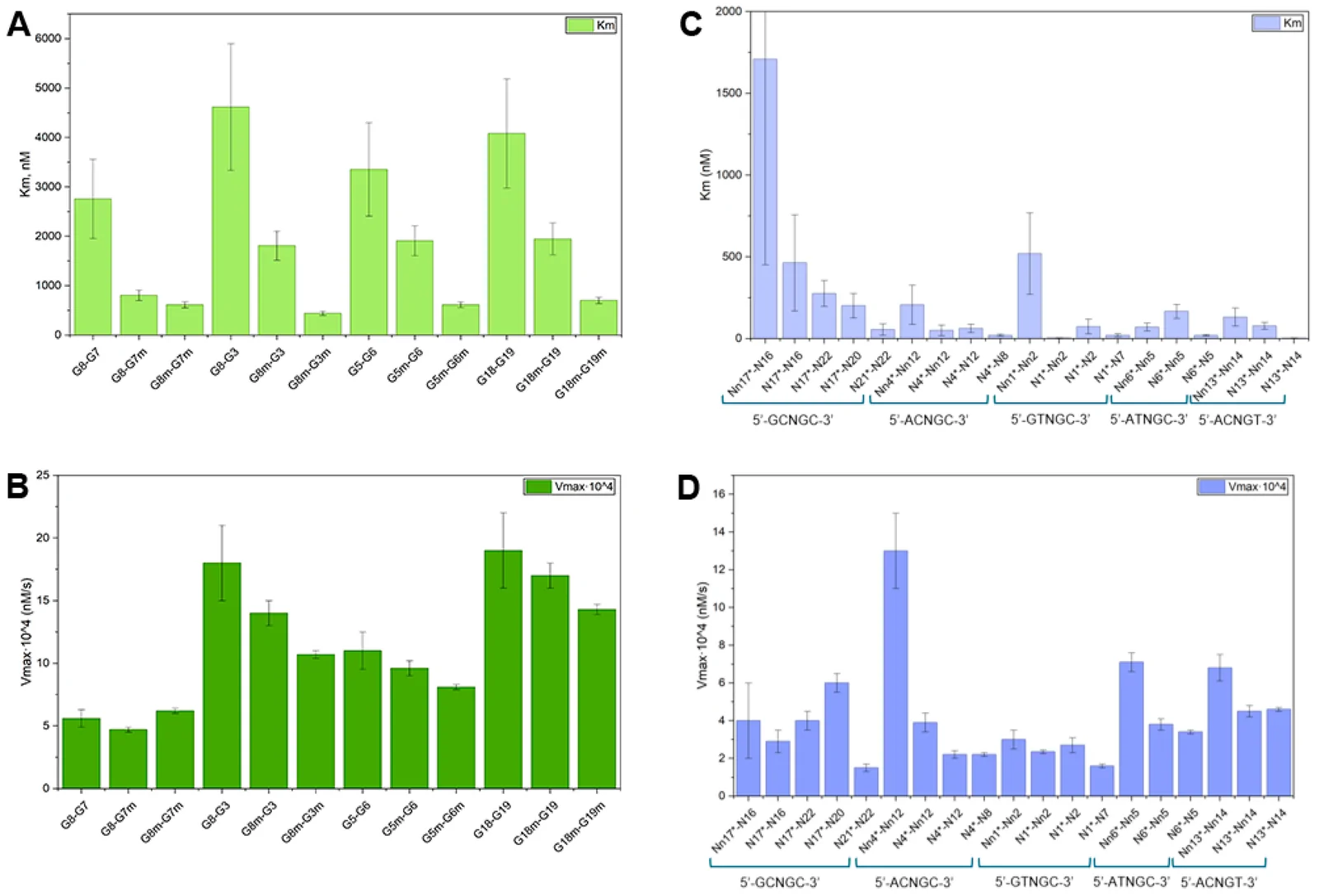
Effect of methylation of oligonucleotide substrate recognition sites on the catalytic parameters K_M_ (**A**,**C**) and V_max_ (**B**,**D**) of restriction endonucleases *Gla*I (**A**,**B**) and *Bls*I (**C**,**D**). *—corresponds to the probes.

Michaelis–Menten constants of *Gla*I restriction endonuclease were determined for the first time and were significantly lower for methylated duplexes than for hemimethylated duplexes, which in turn were lower than for unmethylated duplexes (see Table 3). Our results confirm that the functional affinity of *Gla*I restriction endonuclease for methylated sites is higher than for unmethylated sites (see Figure 9A,B). A decrease in the K_M_ constant in the series G8m-G3m < G8m-G7m < G5m-G6m < G18m-G19m correlates with the literature data [19]. An interesting observation is that for the G8m-G7m duplex, containing four methylated bases, the Michaelis–Menten constant is higher than for G8m-G3m, containing three methylated bases. This may be due to steric hindrance, as two specific sites in the G8m-G7m duplex are located sequentially. This may lead to repulsion between the adjacent site and the enzyme’s active site, or steric hindrance may arise between two enzyme molecules simultaneously attempting to bind adjacent specific sites. V_max_ values differ slightly, ranging from 4.5·10^−4^ to 19.5·10^−4^ nM/s for different substrates, indicating that the reaction efficiency depends primarily on the enzyme–substrate complex formation constant. 

For the *Bls*I endonuclease, it is necessary to compare the obtained K_M_ and V_max_ values depending not only on the number of 5-methylcytosines but also on the sequence of the recognition sites, since its recognition site has a higher degree of degeneracy. Analysis of the kinetic parameters of *Bls*I revealed that corresponding K_M_ constants have the same dependence on the degree of recognition site methylation as those of *Gla*I, except in cases where the functional affinity for asymmetrically methylated duplexes was even lower than for unmethylated ones. Furthermore, increasing the methylation level of the recognition site reduces the error associated with K_M_ calculation (see Table 3 and Table 4). Some differences are observed depending on the sequence of the fully methylated site: K_M_(5′-ACNGT-3′) < K_M_(5′-ACNGC-3′/3′-TGNCG-5′) < K_M_(5′-GTNGC-3′/3′-CANCG-5′) < K_M_(5′-ATNGC-3′) < K_M_(5′-GCNGC-3′), as shown in Figure 9C,D. This may possibly be caused by differences in the GC composition of these sites, which affects their binding to the active center of the enzyme. V_max_ values also vary slightly for different substrates (see Table 4) and regardless of the site sequence or the degree of duplex methylation, which also indicates a predominant dependence of the reaction efficiency on the formation constant of the enzyme–substrate complex.

Values of the kinetic parameters for the methyl-dependent restriction endonucleases are comparable with the known parameters for common representatives of the restriction endonucleases, such as *Bam*HI, *Eco*RI and *Hpa*II. According to the literature data, the values of the K_M_ constants for these enzymes are in range from 0.4 to 4.2 nM, and the V_max_ values vary from 1.5·10^−3^ to 1.6·10^−1^ nM/s [33]. Michaelis–Menten constants of the methyl-dependent restriction endonucleases are close to the constants of the above-mentioned restriction endonucleases in cases of binding to methylated substrates and are much higher than the corresponding values in cases of binding to unmethylated substrates. We suggest this is due to the high specificity of methyl-dependent enzymes to recognition sites containing 5-methylcytosine. At the same time, the maximum velocities are slightly lower than those reported for canonical restriction endonucleases, which can also be attributed to their high site specificity, as the rate-limiting step in this case is the formation of the enzyme–substrate complex.

Thus, it can be concluded that the method for determining kinetic parameters presented in this manuscript not only allows the reliable determination of the K_M_ and V_max_ values for methyl-dependent restriction endonucleases but also allows quantitative characterization of the substrate specificity of these enzymes.

### Limitations

The authors could not determine the exact molar concentration of the enzymes *Gla*I and *Bls*I in the commercial samples because the manufacturer does not provide this information on the website or in the product data sheet and refuses to disclose it separately (however, this is common practice for commercial enzymes). At the same time, we do not consider it rational to standardize the specific activity to units of enzyme activity in 1 mL, which are provided by manufacturer of the restriction endonucleases. Therefore, we could not calculate the V_max_ values by normalizing it to the enzyme molar concentration. However, since all experiments we carried out using enzyme samples with the same production dates and lot numbers, we suggest this did not introduce bias into the analysis and processing of experimental results.

## 5. Conclusions

In this study, we have determined—for the first time—the kinetic parameters of the methyl-dependent restriction endonucleases *Gla*I and *Bls*I using probes containing fluorescent label and quencher. Previously, similar studies were conducted exclusively by electrophoretic methods using radioactive labels (γ-^32^P), as reflected in numerous papers. Values of Michaelis–Menten constants were determined in the range of 400–5000 nM and 2.4–2316 nM for *Gla*I and *Bls*I, respectively. Values of V_max_ were in the ranges of (5–18)·10^−4^ nM/s and (1.4–18)·10^−4^ nM/s for *Gla*I and *Bls*I, respectively. These data also demonstrate high consistency with the literature data and support the hypothesis that the K_M_ values for methyl-dependent restriction endonucleases are lower for fully methylated substrates and higher for unmethylated ones, indicating differences in the active site’s affinity to a specific substrate. Our results confirm that the proposed method can be used as an alternative to traditional approaches. Moreover, the method used is less resource-intensive and offers increased environmental safety compared to radioisotope methods.

## Figures and Tables

**Figure 6:**
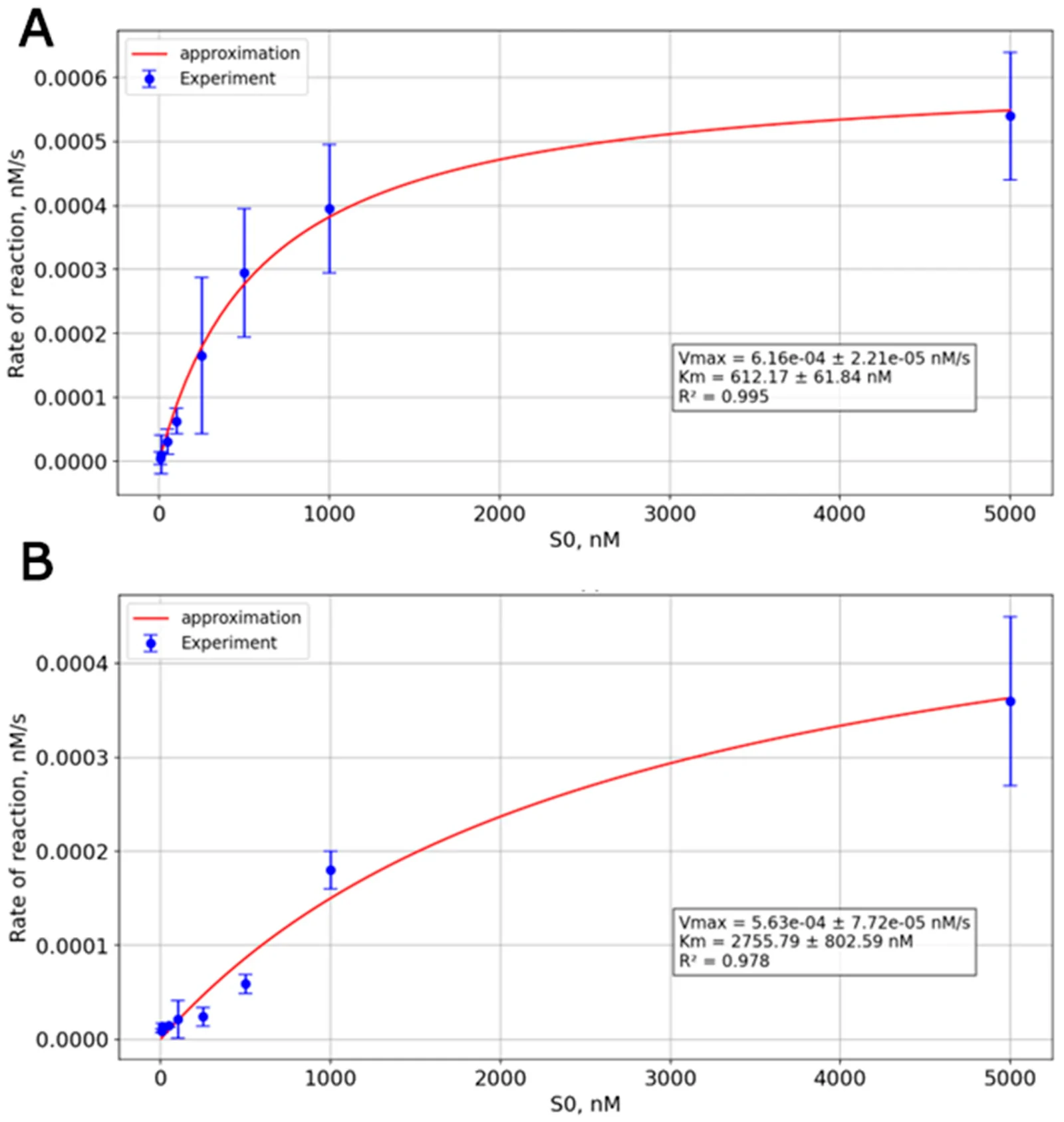
Dependency of the initial velocities on the initial substrate concentration for *Gla*I; duplexes G8m-G7m (**A**) and G8-G7 (**B**) after the approximation by the least squares method.

**Table 1:** Sequences of oligonucleotides used for *Gla*I analysis.

Oligonucleotide Name	Oligonucleotide Sequence
G7m ^1^	5′-TTTTCGM^2^CGMCTGACG-3′
G7	5′-TTTTCGCGCTGACG-3′
G3m	5′-TTTTCGMCGCTGACG-3′
G3	5′-TTTTCGCGCTGACG-3′
G19m	5′-CGTCAAMCGCGAAAA-3′
G19	5′-CGTCAACGCGAAAA-3′
G6m	5′-CGTCAGCGMCGAAAA-3′
G6	5′-CGTCAGCGCGAAAA-3′
G8m *	5′-FAM-CGTCAGMCGMCGAAAA-BHQ1-3′
G18m *	5′-FAM-TTTTCGMCGTTGACG-BHQ1-3′
G5m *	5′-FAM-TTTTCGCGMCTGACG-BHQ1-3′
G8 *	5′-FAM-CGTCAGGGAAAA-BHQ1-3′
G18 *	5′-FAM-TTTTCGGTTGACG-BHQ1-3′
G5 *	5′-FAM-TTTTCGCGTGACG-BHQ1-3′

^1^ m = methylated oligonucleotide; ^2^ M = 5-methyl cytosine; * = probe; FAM—carboxyfluorescein; BHQ—quencher.

**Table 2:** Sequences of oligonucleotides used for *Bls*I analysis.

Oligonucleotide Name	Oligonucleotide Sequence
N21 *	5′-FAM-GCTTGTACTTTAGM^1^CGGMCATTGATTCTCACCACG-BHQ1-3′
N17 *	5′-FAM-GCTTGTACTTTAGMCGGCATTGATTCTCACCACG-BHQ1-3′
N22	5′-CGTGGTGAGAATCAATGMCCGMCTAAAGTACAAGC-3′
N20	5′-CGTGGTGAGAATCAATGCCGMCTAAAGTACAAGC-3′
N16	5′-CGTGGTGAGAATCAATGCCGCTAAAGTACAAGC-3′
Nn17 *	5′-FAM-GCTTGTACTTTAGCGGCATTGATTCTCACCACG-BHQ1-3′
N4 *	5′-FAM-GGGAAAAGMCTGTAAAAGAGGAAAGGG-BHQ1-3′
N8	5′-CCCTTTCCTCTTTTAMCAGMCTTTTCCC-3′
N12	5′-CCCTTTCCTCTTTTAMCAGCTTTTCCC-3′
Nn4 *	5′-FAM-GGGAAAAGCTGTAAAAGAGGAAAGGG-BHQ1-3′
Nn12	5′-CCCTTTCCTCTTTTACAGCTTTTCCC-3′
N1 *	5′-FAM-CCCTTTCCTCTTTTGTAGMCTTTTCCC-BHQ1-3′
N7	5′-GGGAAAAGMCTAMCAAAAGAGGAAAGGG-3′
N2	5′-GGGAAAAGMCTACAAAAGAGGAAAGGG-3′
Nn2	5′-GGGAAAAGCTACAAAAGAGGAAAGGG-3′
Nn1 *	5′-FAM-CCCTTTCCTCTTTTGTAGCTTTTCCC-BHQ1-3′
N6 *	5′-FAM-GGGAAAAGMCTATAAAAGAGGAAAGGG-BHQ1-3′
N5	5′-CCCTTTCCTCTTTTATAGMCTTTTCCC-3′
Nn6 *	5′-FAM-GGGAAAAGCTATAAAAGAGGAAAGGG-BHQ1-3′
Nn5	5′-CCCTTTCCTCTTTTATAGCTTTTCCC-3′
N13 *	5′-FAM-GCTTGTACTTTGAMCAGTATTGATTCTCACCACG-BHQ1-3′
N14	5′-CGTGGTGAGAATCAATAMCTGTCAAAGTACAAGC-3′
Nn13 *	5′-FAM-GCTTGTACTTTGACAGTATTGATTCTCACCACG-BHQ1-3′
Nn14	5′-CGTGGTGAGAATCAATACTGTCAAAGTACAAGC-3′

^1^ M = 5-methyl cytosine; * = probes, containing 5′-FAM (carboxyfluorescein) and 3′-BHQ1.

**Table 3:** Kinetic parameters of *Gla*I were determined by the processing of the fluorescence data using the least squares method.

Duplex	K_M_·10^−2^, nM	V_max_·10^−4^, nM/s
*G8-G7	28 ± 8 *	5.6 ± 0.7
*G8-G7m	8 ± 1	4.7 ± 0.2
*G8m-G7m	6.1 ± 0.6	6.2 ± 0.2
*G8-G3	50 ± 10	18 ± 3
*G8m-G3	18 ± 3	14 ± 1
*G8m-G3m	4.4 ± 0.4	10.7 ± 0.3
*G5-G6	34 ± 9	11 ± 2
*G5m-G6	19 ± 3	9.6 ± 0.6
*G5m-G6m	6.1 ± 0.6	8.1 ± 0.2
*G18-G19	40 ± 1	19 ± 3
*G18m-G19	19 ± 3	17 ± 1
*G18m-G19m	7 ± 0.6	14.3 ± 0.4

Values ± standard errors of nonlinear regression fit are presented; *—corresponds to the probes.

**Table 4:** Kinetic parameters of *Bls*I were determined from fluorescence data by the least squares method.

Duplex	K_M_·10^−2^, nM	V_max_·10^−4^, nM/s
Recognition site 5′-GCNGC-3′		
Nn17*-N16	20 ± 10	4 ± 2
N17*-N16	5 ± 3	2.9 ± 0.6
N17*-N22	2.7 ± 0.8	4.0 ± 0.4
N17*-N20	2 ± 0.8	6.0 ± 0.5
N21*-N22	0.5 ± 0.3	1.5 ± 0.2
Recognition site 5′-ACNGC-3′/3′-TGNCG-5′		
Nn4*-Nn12	2 ± 1	13 ± 2
N4*-Nn12	0.5 ± 0.3	3.9 ± 0.5
N4*-N12	0.6 ± 0.2	2.2 ± 0.2
N4*-N8	0.2 ± 0.07	2.2 ± 0.1
Recognition site 5′-GTNGC-3′/3′-CANCG-5′		
Nn1*-Nn2	5 ± 2	3.0 ± 0.5
N1*-Nn2	0.03 ± 0.01	2.4 ± 0.1
N1*-N2	0.7 ± 0.4	2.7 ± 0.4
N1*-N7	0.2 ± 0.09	1.6 ± 0.1
Recognition site 5′-ATNGC-3′		
Nn6*-Nn5	0.7 ± 0.2	7.1 ± 0.5
N6*-Nn5	2 ± 0.4	3.8 ± 0.3
N6*-N5	0.19 ± 0.05	3.4 ± 0.1
Recognition site 5′-ACNGT-3′		
Nn13*-Nn14	1.3 ± 0.5	6.8 ± 0.7
N13*-Nn14	0.8 ± 0.2	4.5 ± 0.3
N13*-N14	0.025 ± 0.008	4.6 ± 0.1

Values ± standard errors of nonlinear regression fit are presented; *—corresponds to the probes.

## Data Availability

All the data corresponding to the results presented in the Article are listed in the Supplementary Materials, the Python scripts were uploaded within the manuscript submission. Any additional data corresponding to the manuscript might be provided upon direct request to the corresponding author.

## Supplementary Materials

The following supporting information can be downloaded at: https://www.mdpi.com/article/10.3390/biom16081217/s1, Table S1. Concentrations and volumes of the solutions. Table S2. Composition of reaction mixtures for *Gla*I analysis. Table S3. Composition of reaction mixtures for *Bls*I analysis. Figure S1. Linear dependence of the initial velocity on the substrate concentration. Figure S2. Approximation for duplex G8-G7. Figure S3. Approximation for duplex G8-G7m. Figure S4. Approximation for duplex G8m-G7m. Figure S5. Approximation for duplex G8-G3. Figure S6. Approximation for duplex G8m-G3. Figure S7. Approximation for duplex G8m-G3m. Figure S8. Approximation for duplex G5-G6. Figure S9. Approximation for duplex G5m-G6. Figure S10. Approximation for duplex G5m-G6m. Figure S11. Approximation for duplex G18-G19. Figure S12. Approximation for duplex G18m-G19. Figure S13. Approximation for duplex G18m-G19m. Figure S14. Approximation for duplex Nn17-N16. Figure S15. Approximation for duplex N17*-N16. Figure S16. Approximation for duplex N17*-N22. Figure S17. Approximation for duplex N17*-N20. Figure S18. Approximation for duplex N21*-N22. Figure S19. Approximation for duplex Nn4*-Nn12. Figure S20. Approximation for duplex N4*-Nn12. Figure S21. Approximation for duplex N4*-N12. Figure S22. Approximation for duplex N4*-N8. Figure S23. Approximation for duplex Nn1*-Nn2. Figure S24. Approximation for duplex N1*-Nn2. Figure S25. Approximation for duplex N1*-N2. Figure S26. Approximation for duplex N1*-N7. Figure S27. Approximation for duplex Nn6*-Nn5. Figure S28. Approximation for duplex N6*-Nn5. Figure S29. Approximation for duplex N6*-N5. Figure S30. Approximation for duplex Nn13*-Nn14. Figure S31. Approximation for duplex N13*-Nn14. Figure S32. Approximation for duplex N13*-N14.

## Abbreviations

The following abbreviations are used in this manuscript:

FRET	Förster resonance energy transfer
K_M_	Michaelis–Menten constant
V_max_	Maximal reaction velocity
DMSO	Dimethyl sulfoxide
FAM	6-Carboxyfluorescein
BHQ	Black Hole Quencher
