# Supplementary material for: Fluorescent Analysis of KM and Vmax Values for Methyl-Dependent Restriction Endonucleases
Source: Biomolecules. 2026 Aug 20;16(8):1217. doi: 10.3390/biom16081217 (PMC13510852; doi:10.3390/biom16081217)
Supplement: Supplementary file 1 [file biomolecules-16-01217-s001.zip › biomolecules-4462239-supplementary.pdf]

Supplementary Materials

Table S1. Concentrations and volumes of the solutions.

	**Solution 1**	**Solution 2**	**Solution 3**
Cduplex, nM	5000	500	50
V, µl	40	100	40

Table S2. Composition of reaction mixtures for *Gla*I analysis.

**Sample number:**	**1**	**2**	**3**	**4**	**5**	**6**	**7**	**8**
Buffer B, µl	10	10	10	10	10	10	10	10
DMSO, µl	20	20	20	20	20	20	20	20
GlaI, µl [1]	1	1	1	1	1	1	1	1
milliQ, µl	64	49	59	19	49	59	49	59
duplex, µl	5	20	10	50	20	10	20	10
Cduplex, nM	2500	1000	500	250	100	50	10	5
Duplex solution	Initial – 50 µM		Solution 1 – 5 µM		Solution 2 – 500 nM		Solution 3 – 50 nM	

[1] - 500 a.u./µl.

Table S3. Composition of reaction mixtures for *Bls*I analysis.

**Sample number:**	**1**	**2**	**3**	**4**	**5**	**6**	**7**	**8**
Buffer W, µl	10	10	10	10	10	10	10	10
BlsI, µl [1]	1	1	1	1	1	1	1	1
milliQ, µl	79	69	79	39	69	79	69	79
duplex, µl	10	20	10	50	20	10	20	10
Cduplex, nM	5000	1000	500	250	100	50	10	5
Duplex solution	Initial – 50 µM		Solution 1 – 5 µM		Solution 2 – 500 nM		Solution 3 – 50 nM	

[1] - 500 a.u./µl.

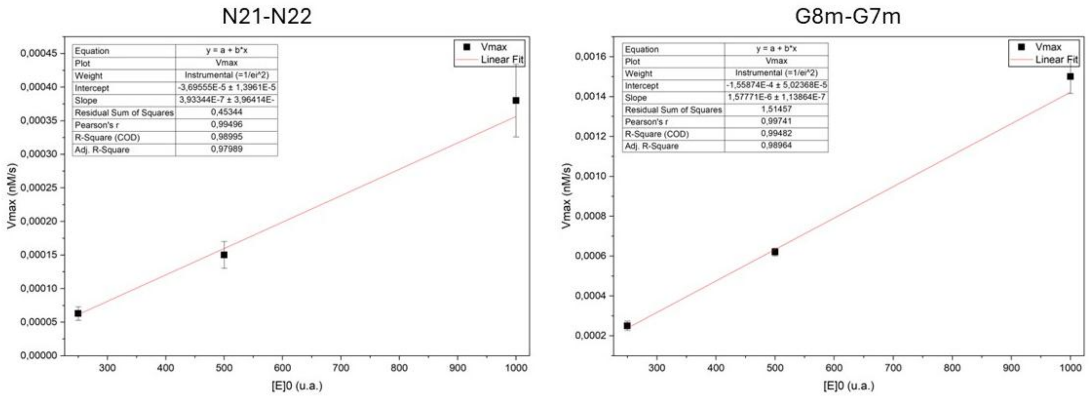

Figure S1. Linear dependence of the initial velocity on the substrate concentration.

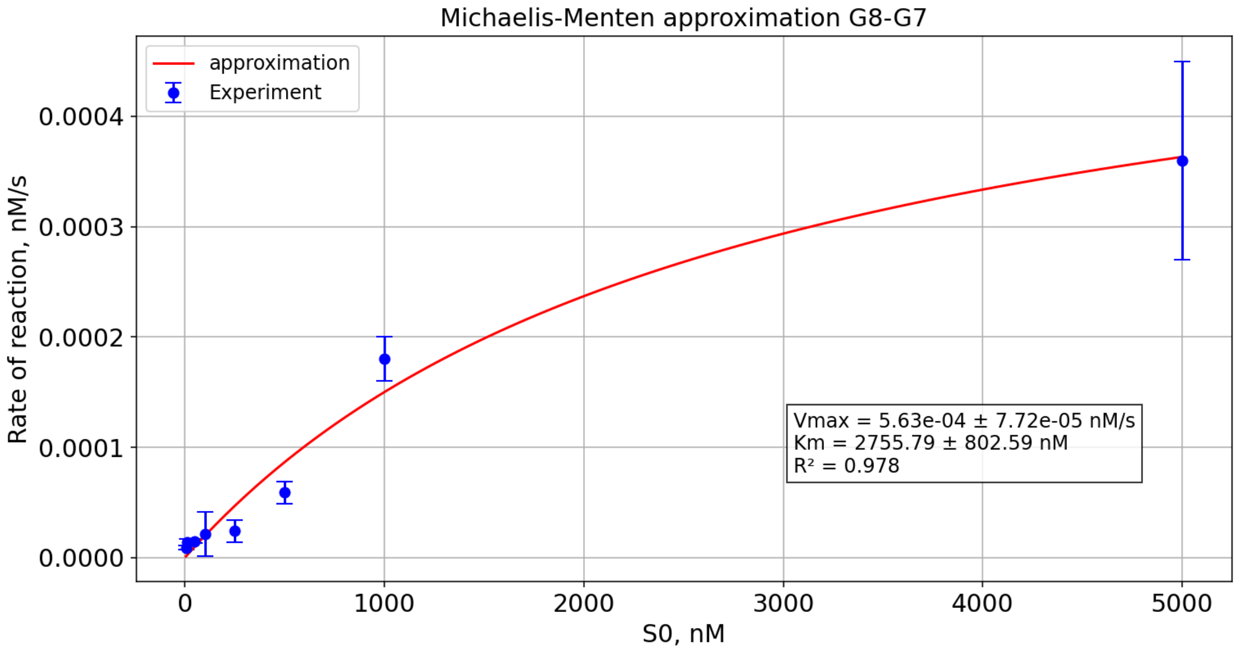

Figure S2. Approximation for duplex G8-G7.

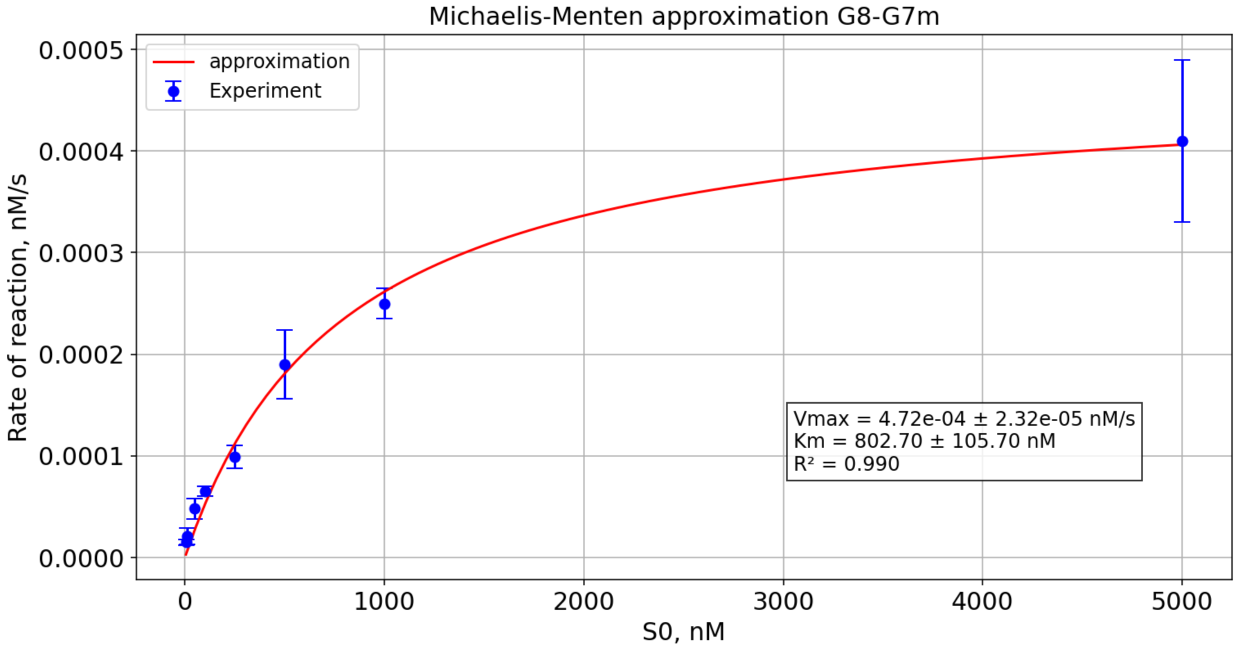

Figure S3. Approximation for duplex G8-G7m.

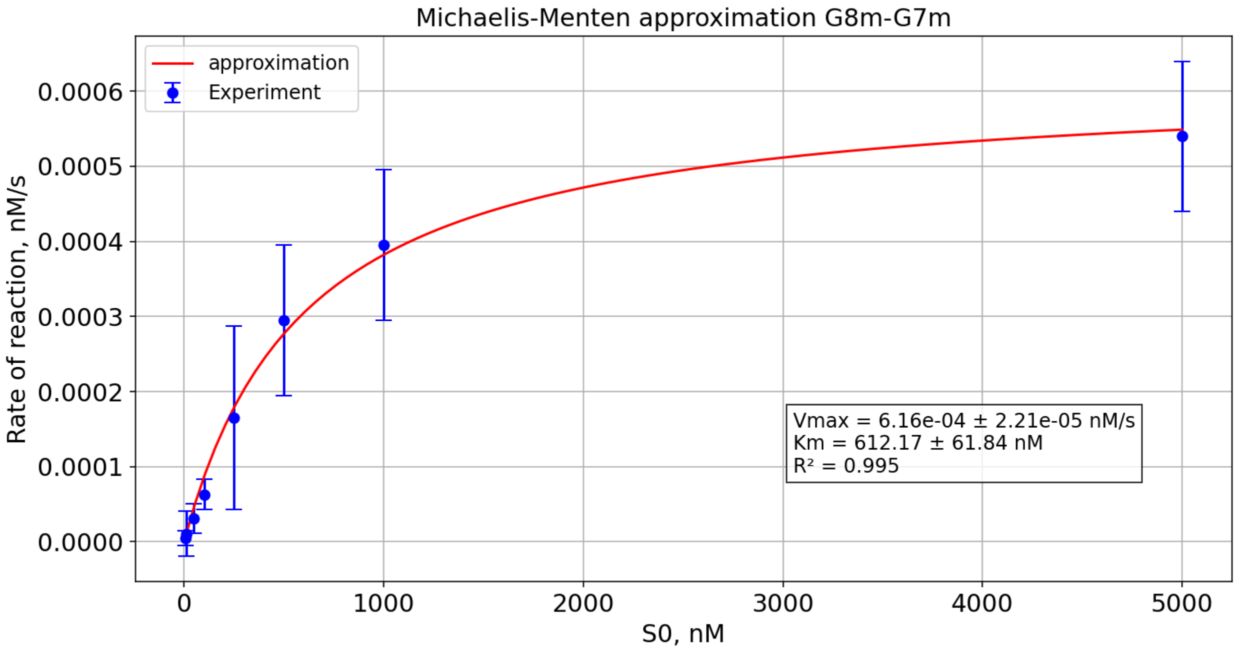

Figure S4. Approximation for duplex G8m-G7m.

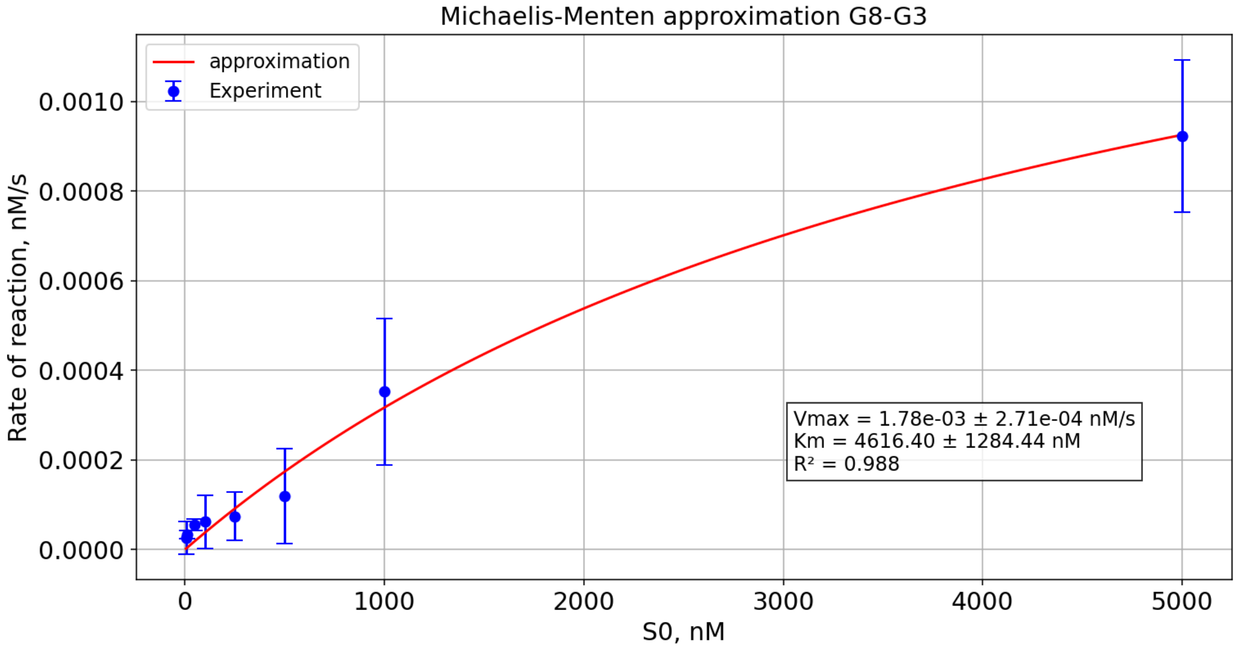

Figure S5. Approximation for duplex G8-G3.

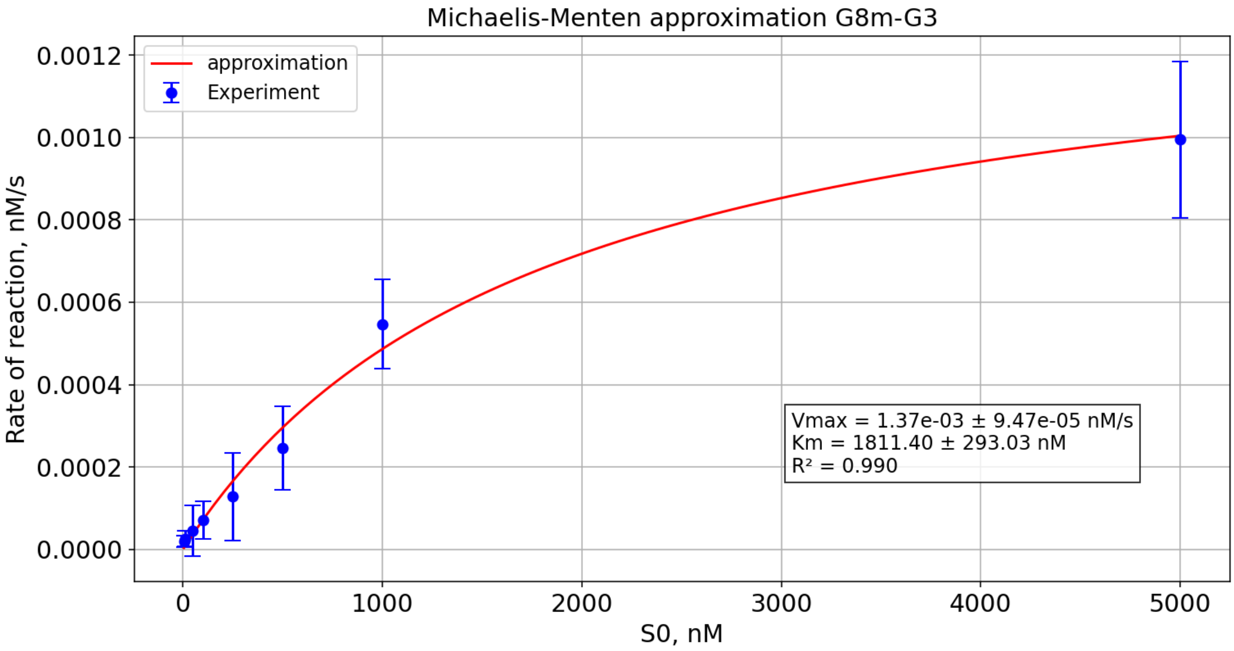

Figure S6. Approximation for duplex G8m-G3.

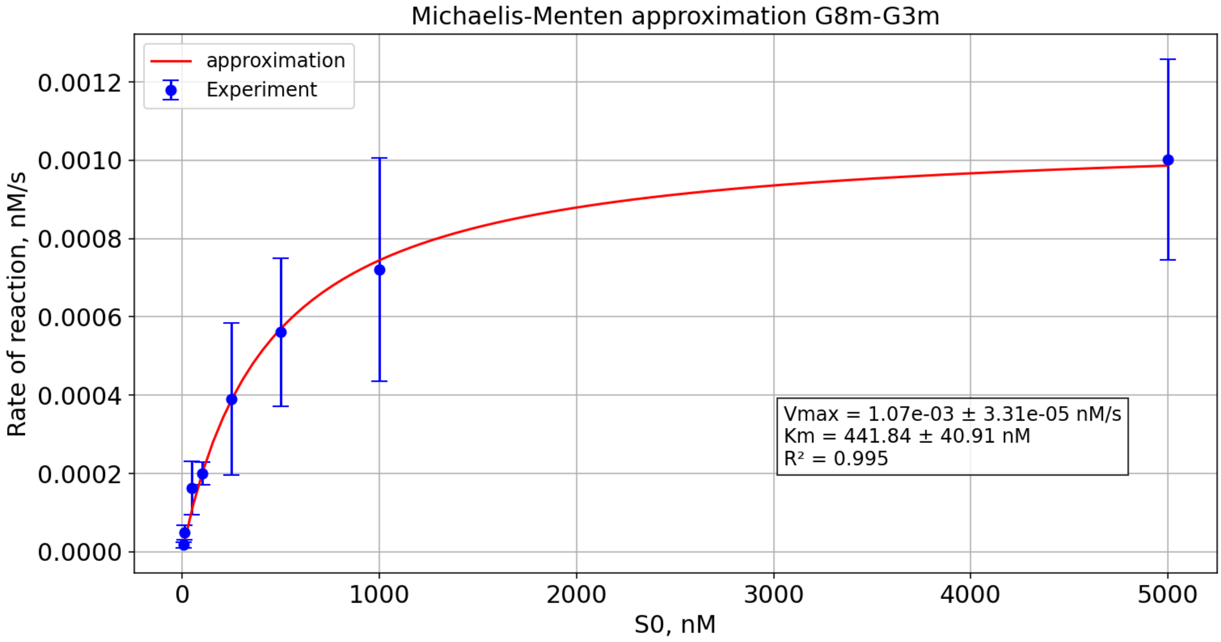

Figure S7. Approximation for duplex G8m-G3m.

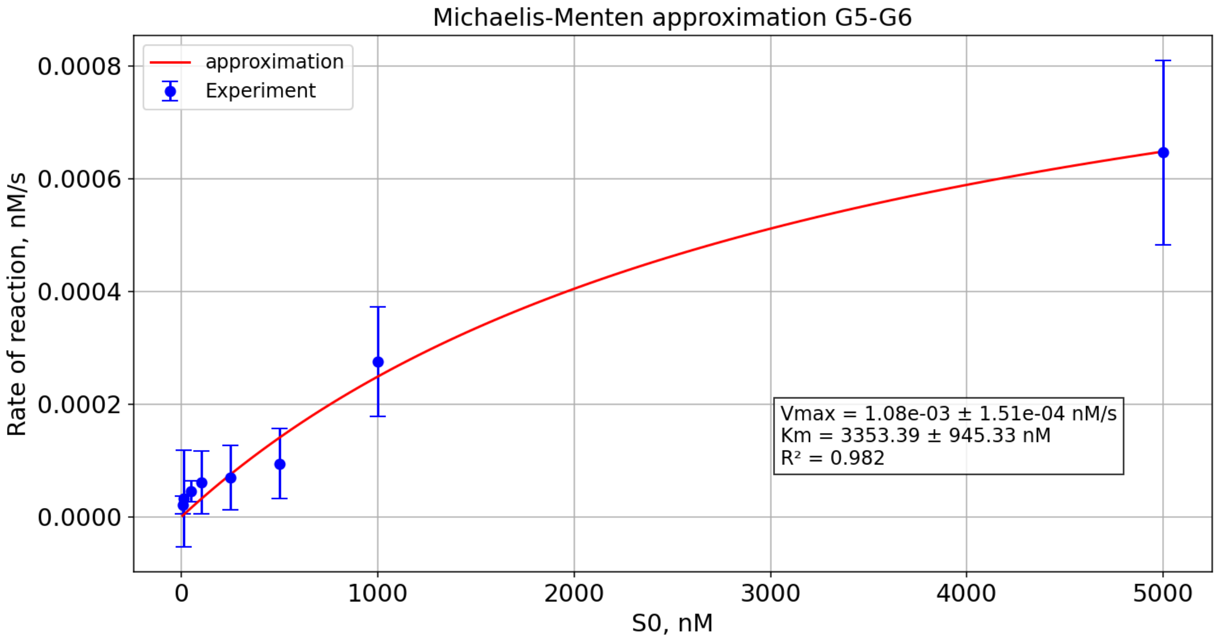

Figure S8. Approximation for duplex G5-G6.

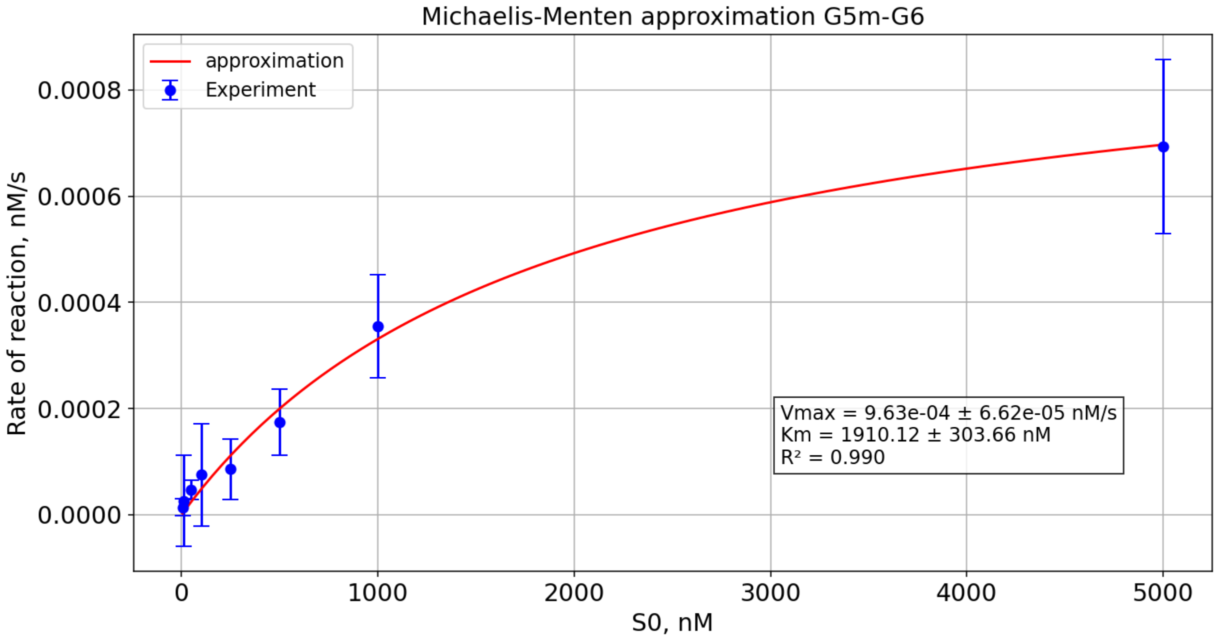

Figure S9. Approximation for duplex G5m-G6

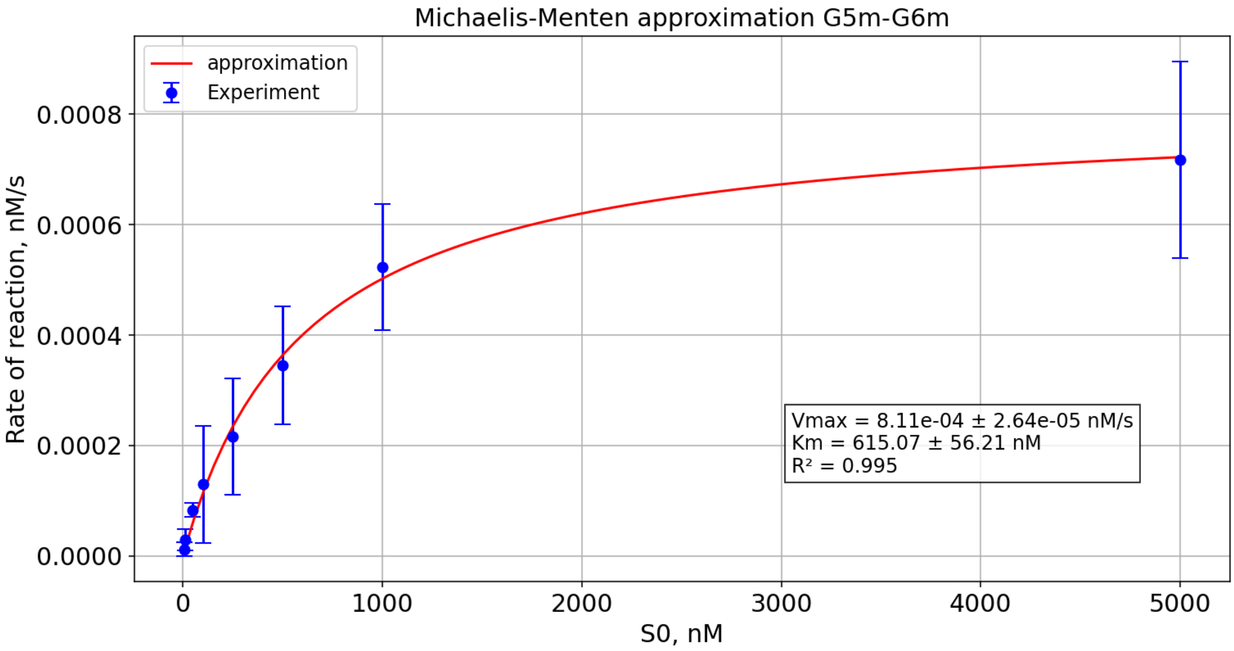

Figure S10. Approximation for duplex G5m-G6m.

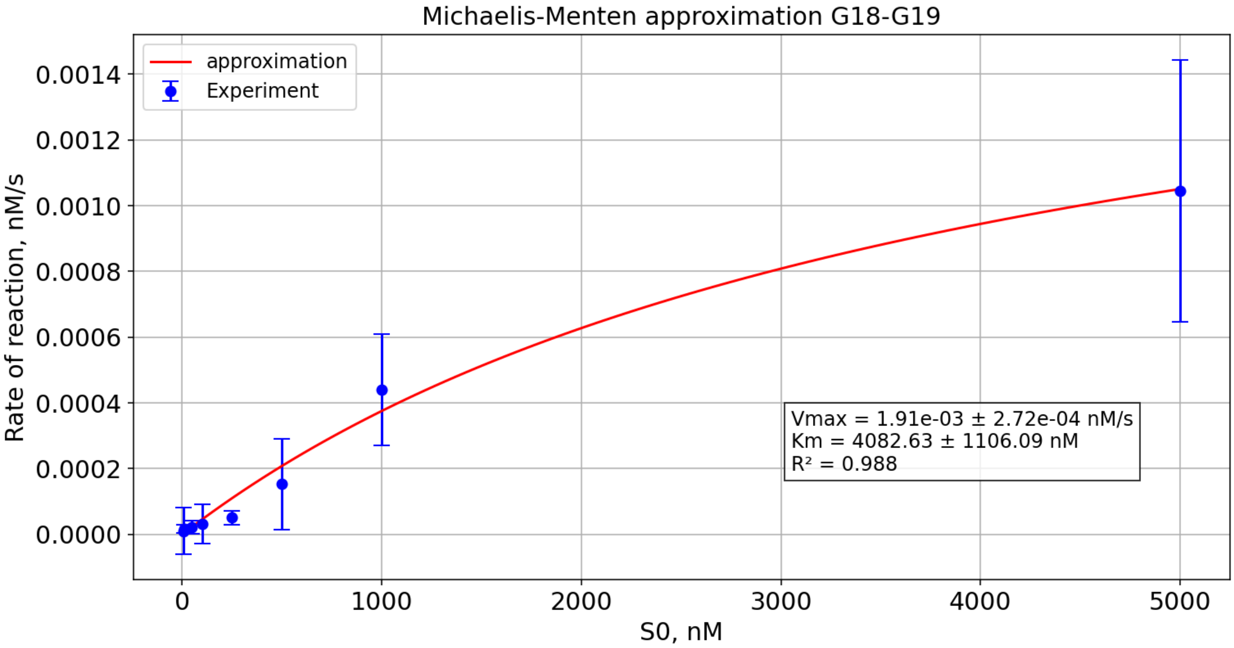

Figure S11. Approximation for duplex G18-G19.

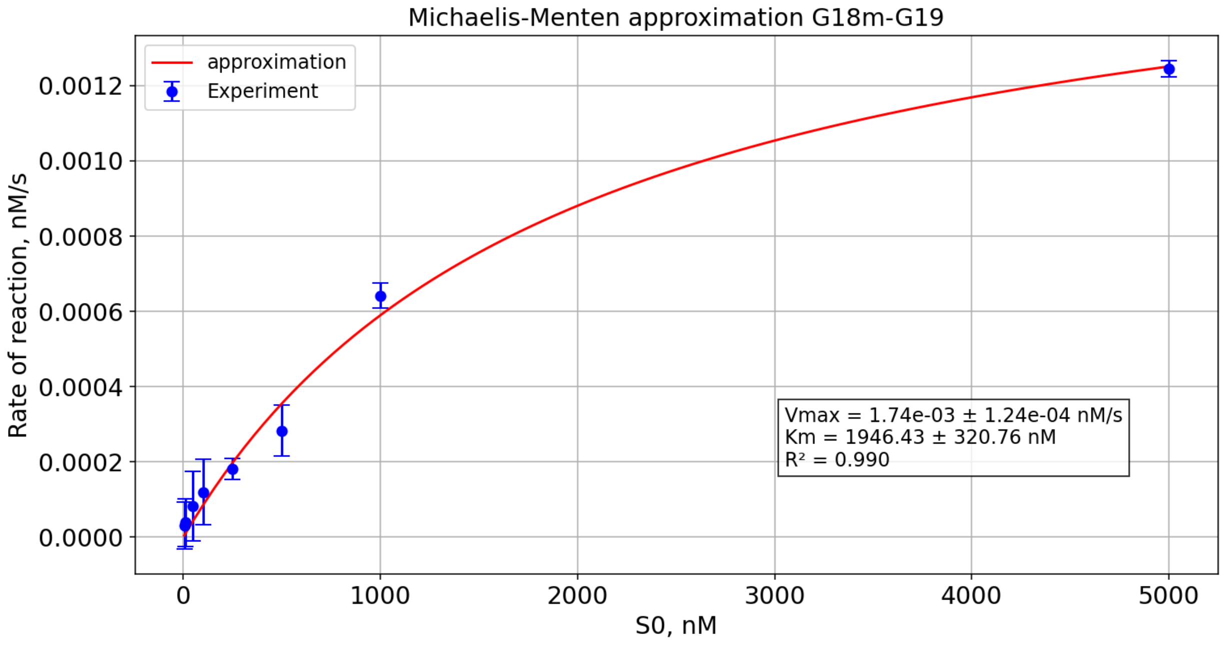

Figure S12. Approximation for duplex G18m-G19.

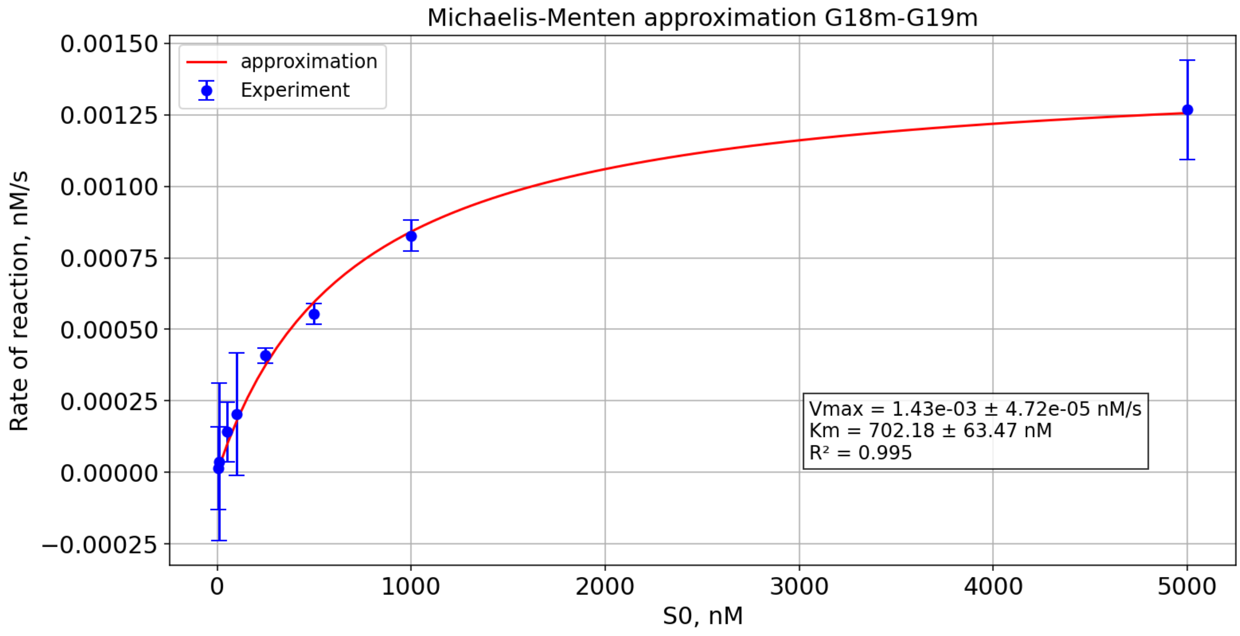

Figure S13. Approximation for duplex G18m-G19m.

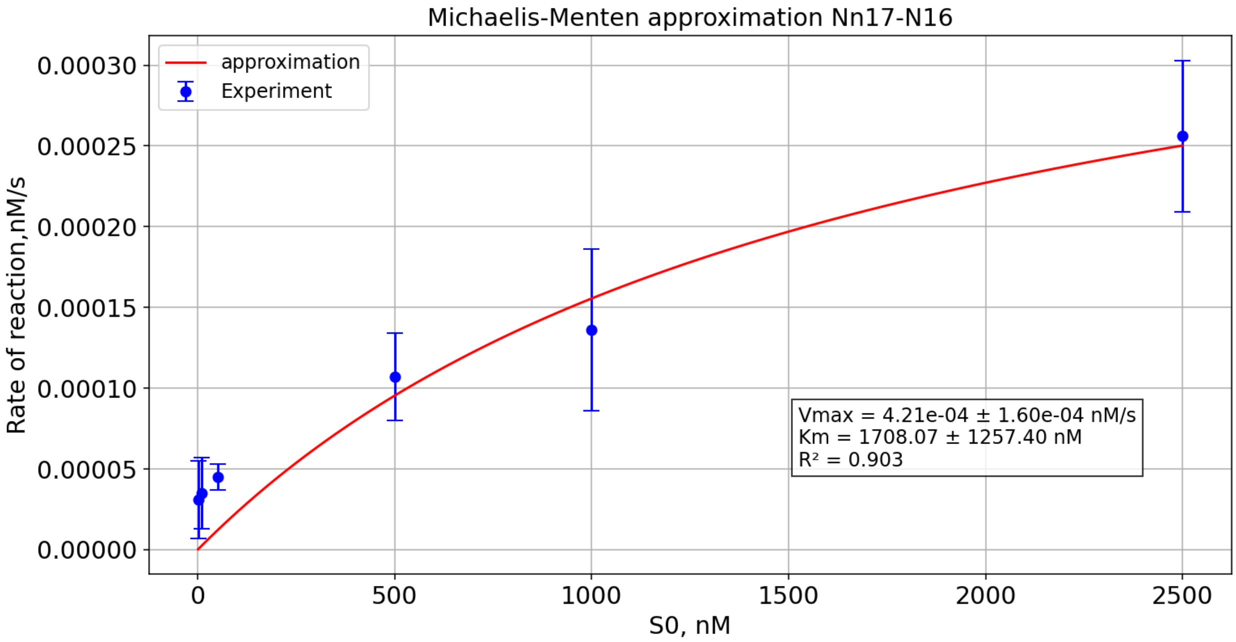

Figure S14. Approximation for duplex Nn17-N16.

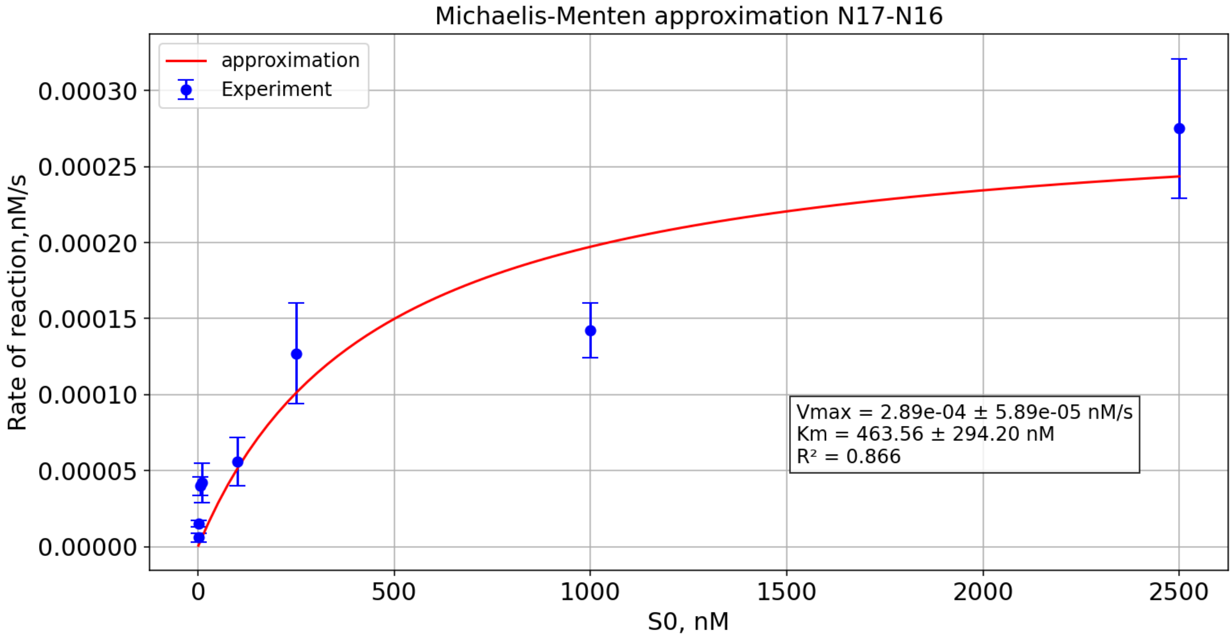

Figure S15. Approximation for duplex N17*-N16.

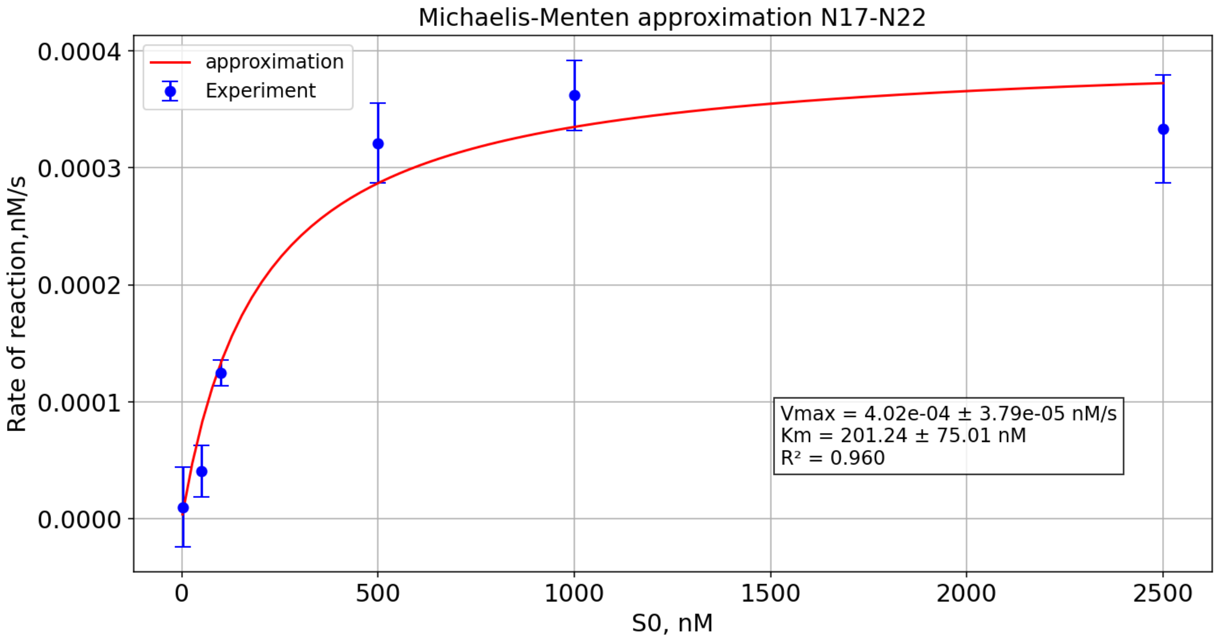

Figure S16. Approximation for duplex N17*-N22.

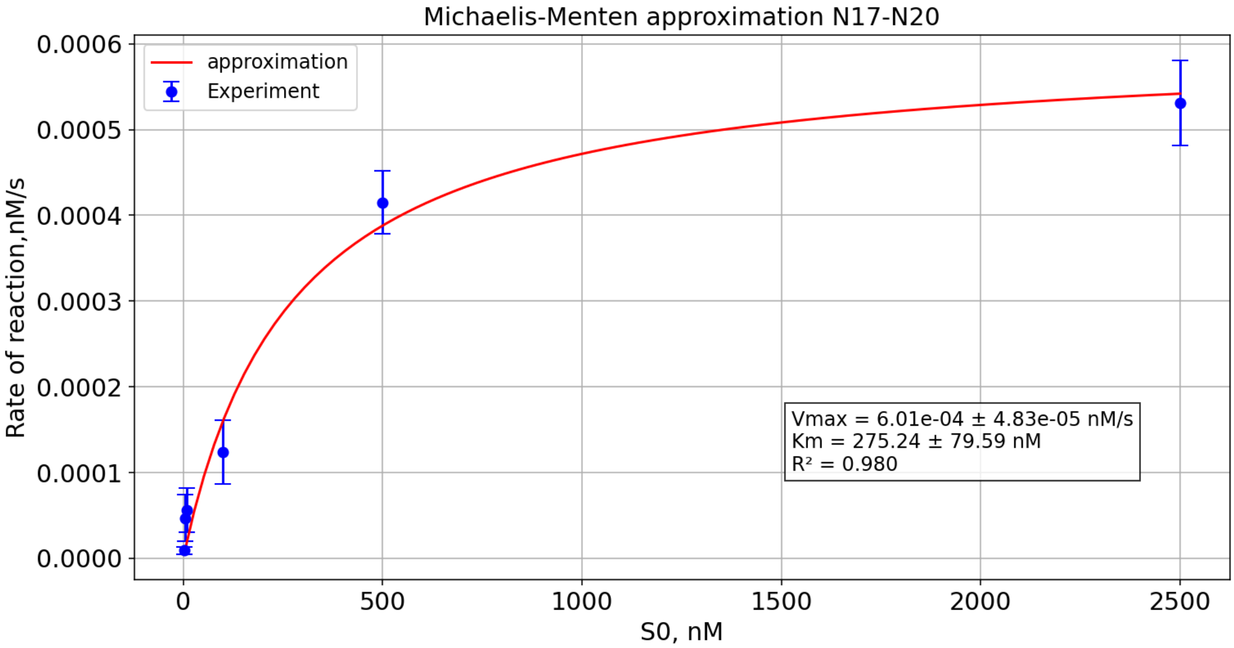

Figure S17. Approximation for duplex N17*-N20.

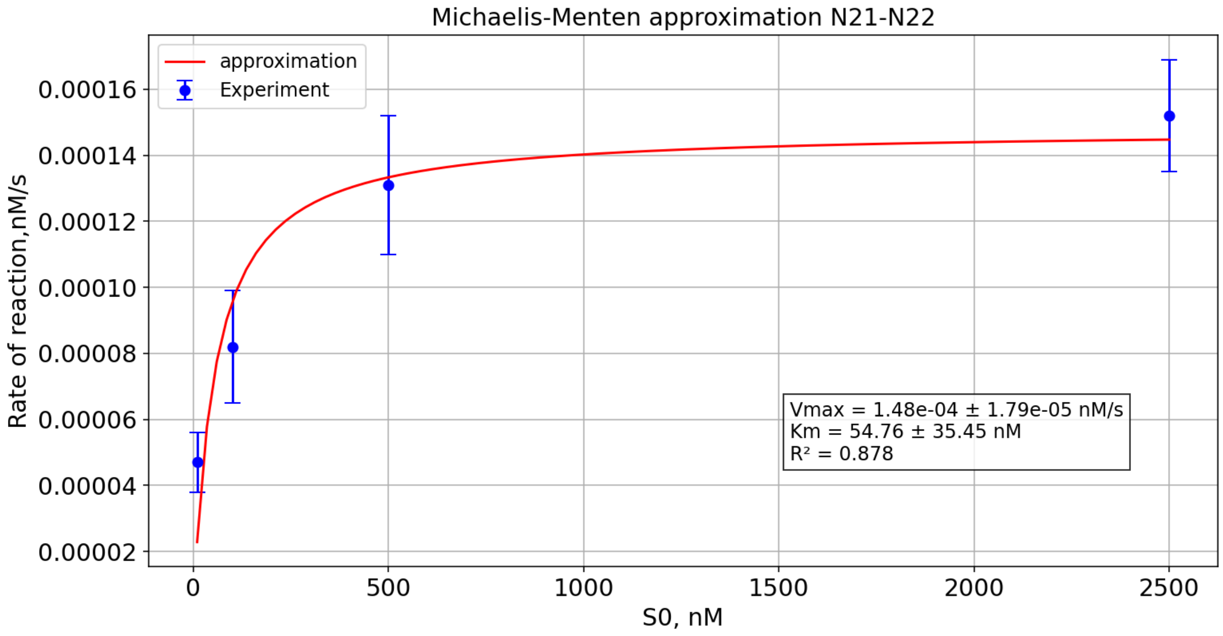

Figure S18. Approximation for duplex N21*-N22.

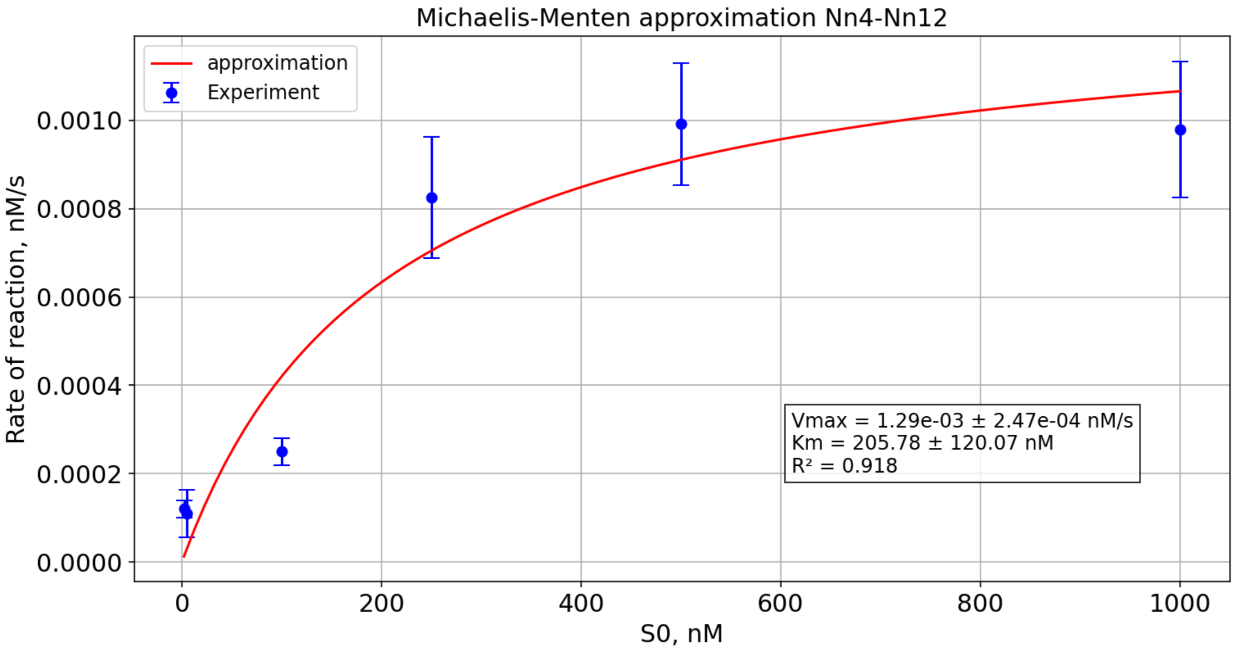

Figure S19. Approximation for duplex Nn4*-Nn12

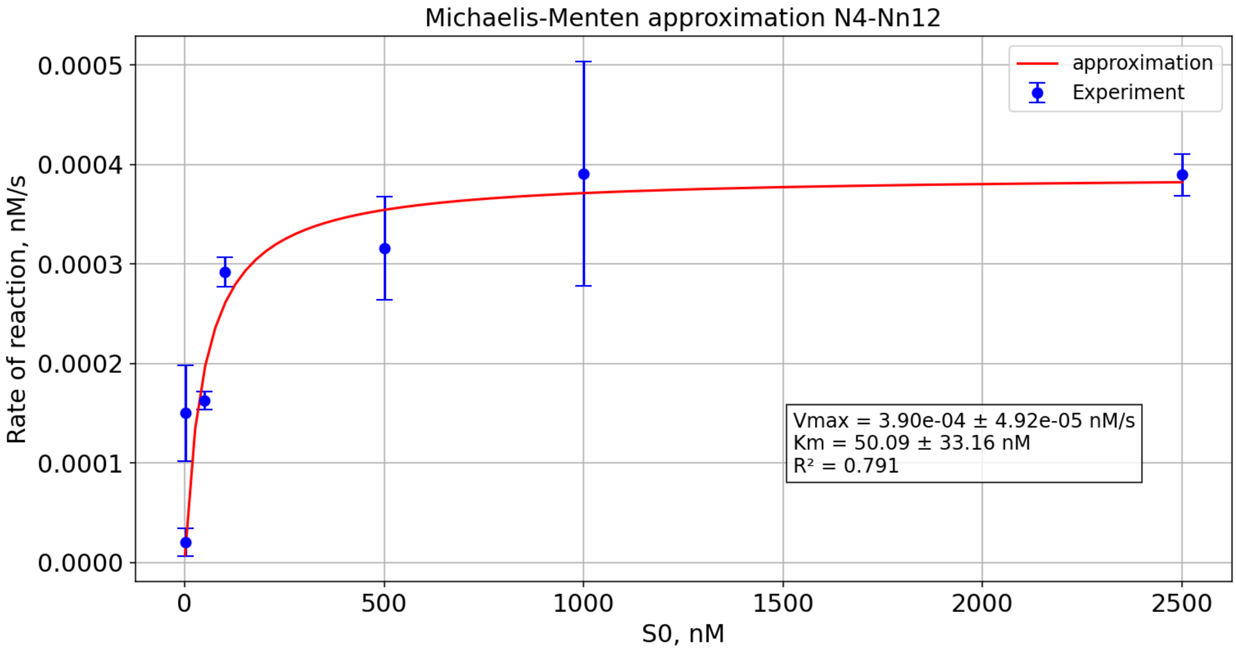

Figure S20. Approximation for duplex N4*-Nn12.

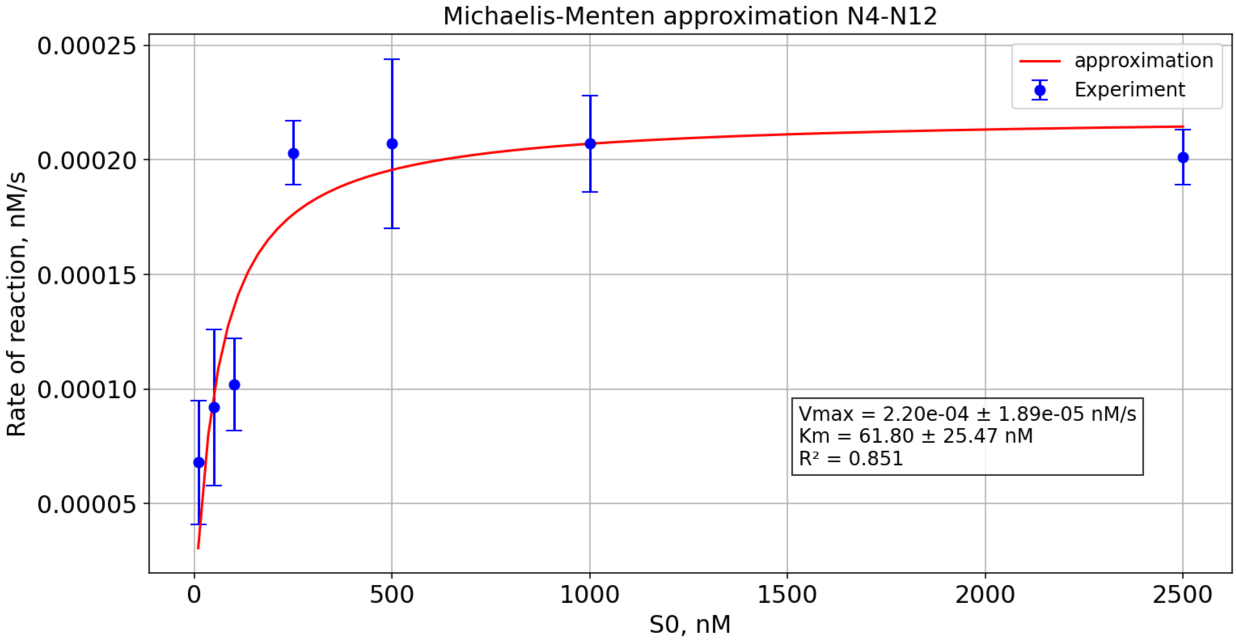

Figure S21. Approximation for duplex N4*-N12.

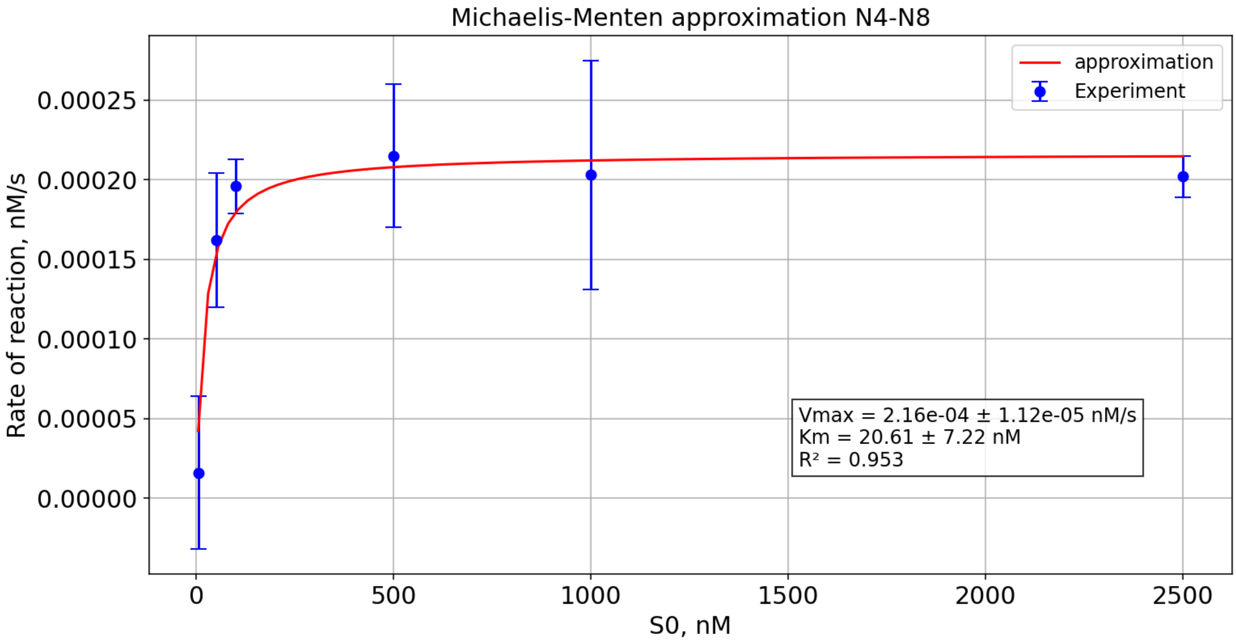

Figure S22. Approximation for duplex N4*-N8.

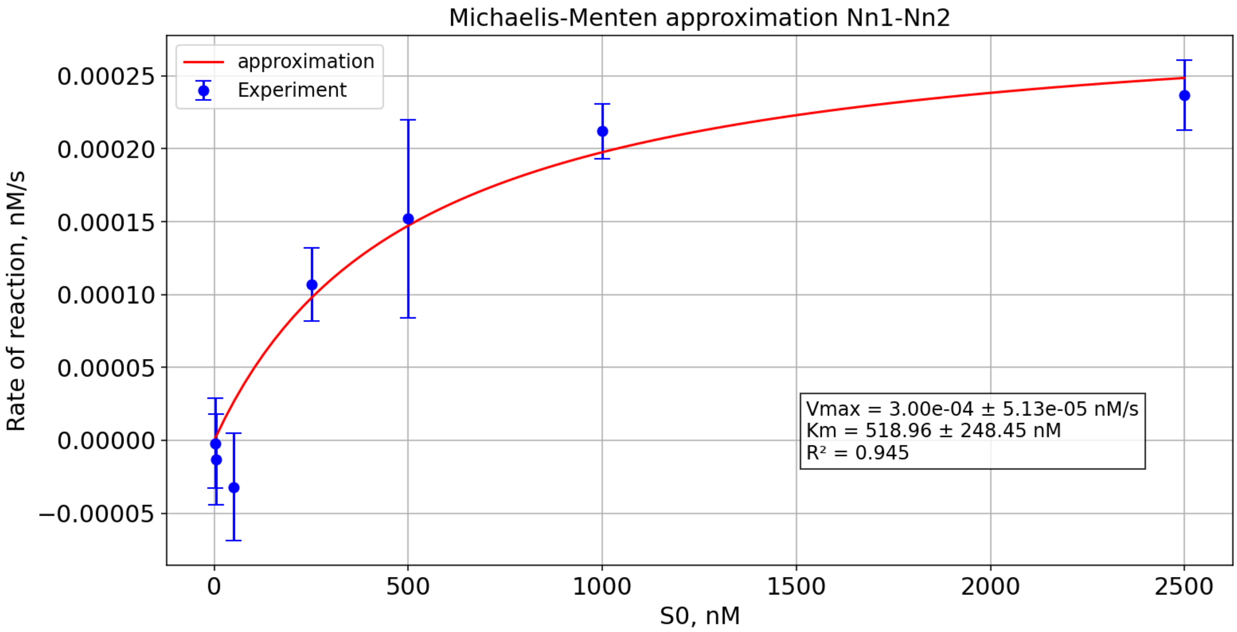

Figure S23. Approximation for duplex Nn1*-Nn2.

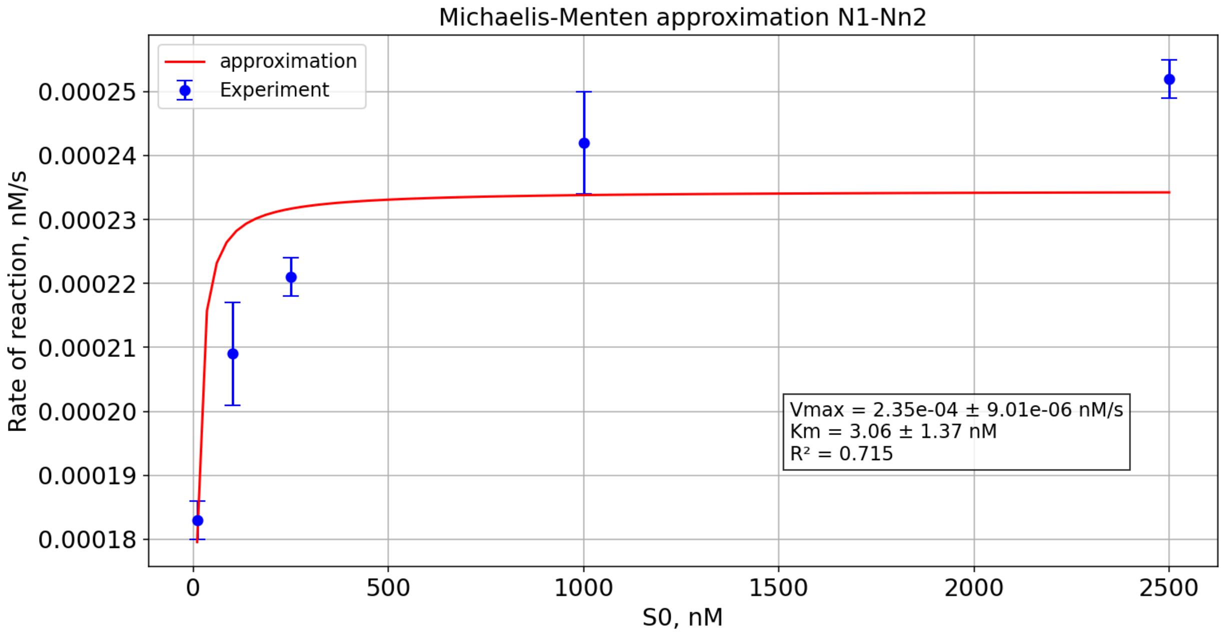

Figure S24. Approximation for duplex N1*-Nn2.

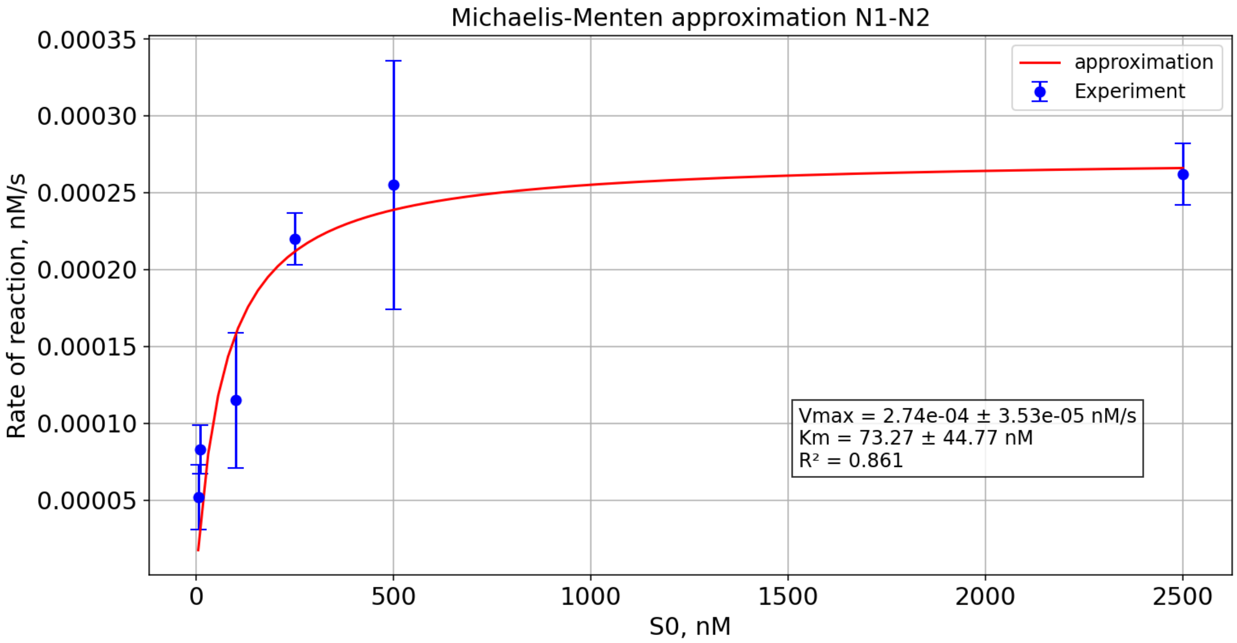

Figure S25. Approximation for duplex N1*-N2.

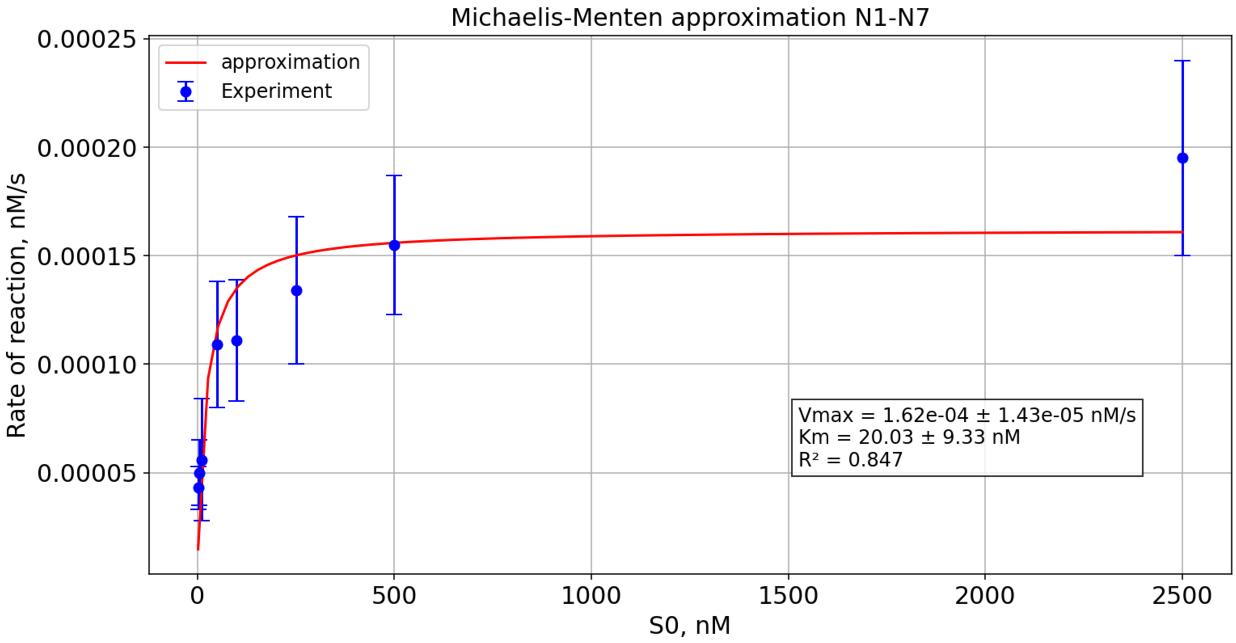

Figure S26. Approximation for duplex N1*-N7.

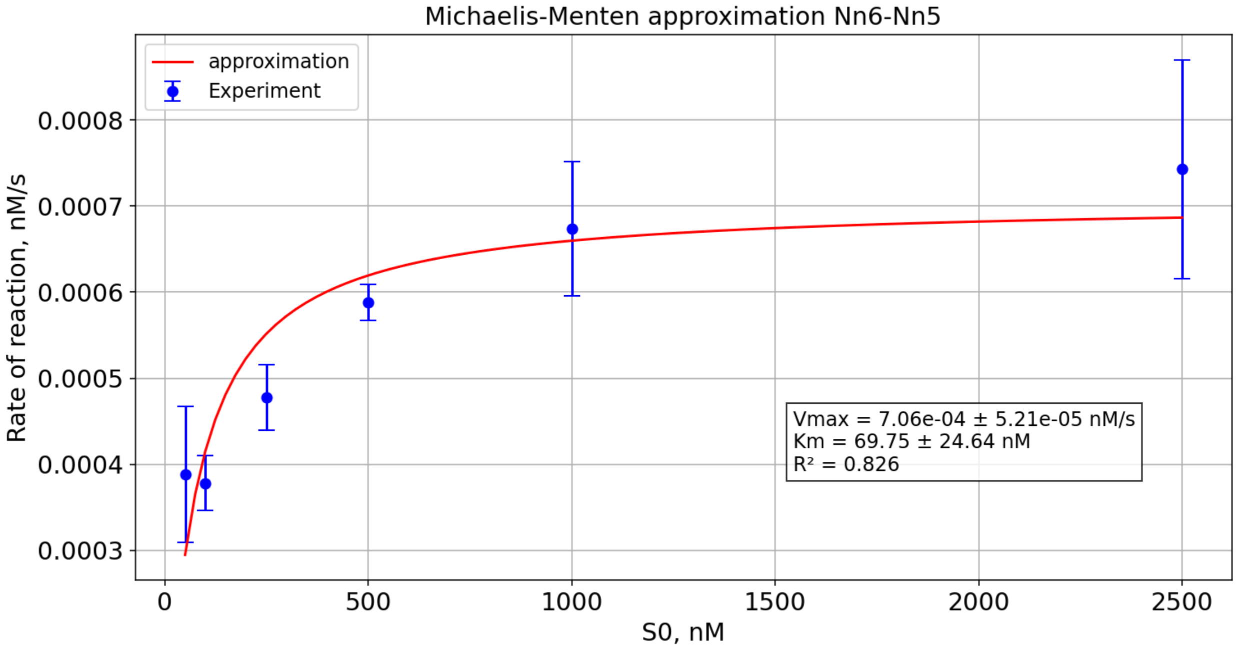

Figure S27. Approximation for duplex Nn6*-Nn5.

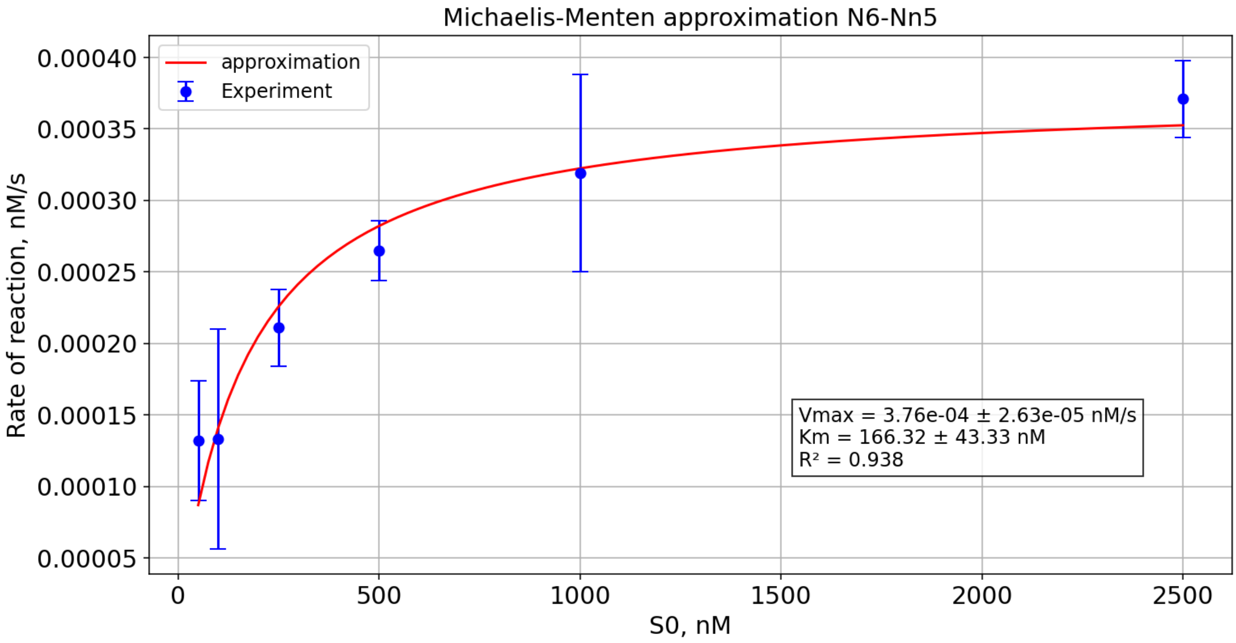

Figure S28. Approximation for duplex N6*-Nn5.

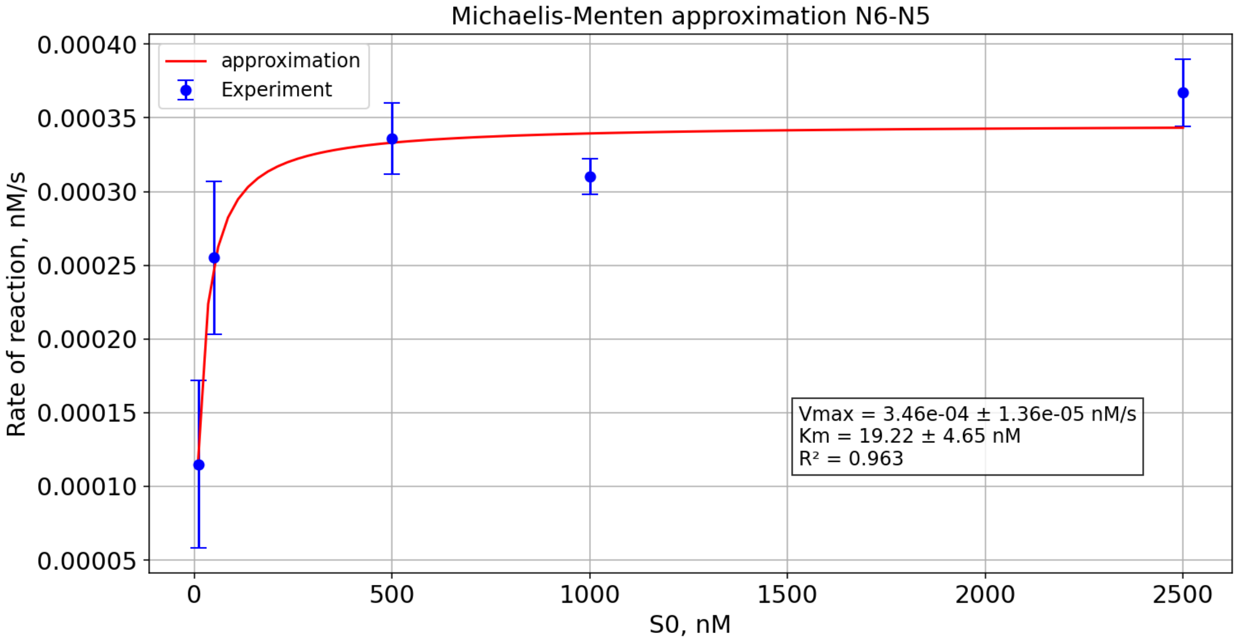

Figure S29. Approximation for duplex N6*-N5.

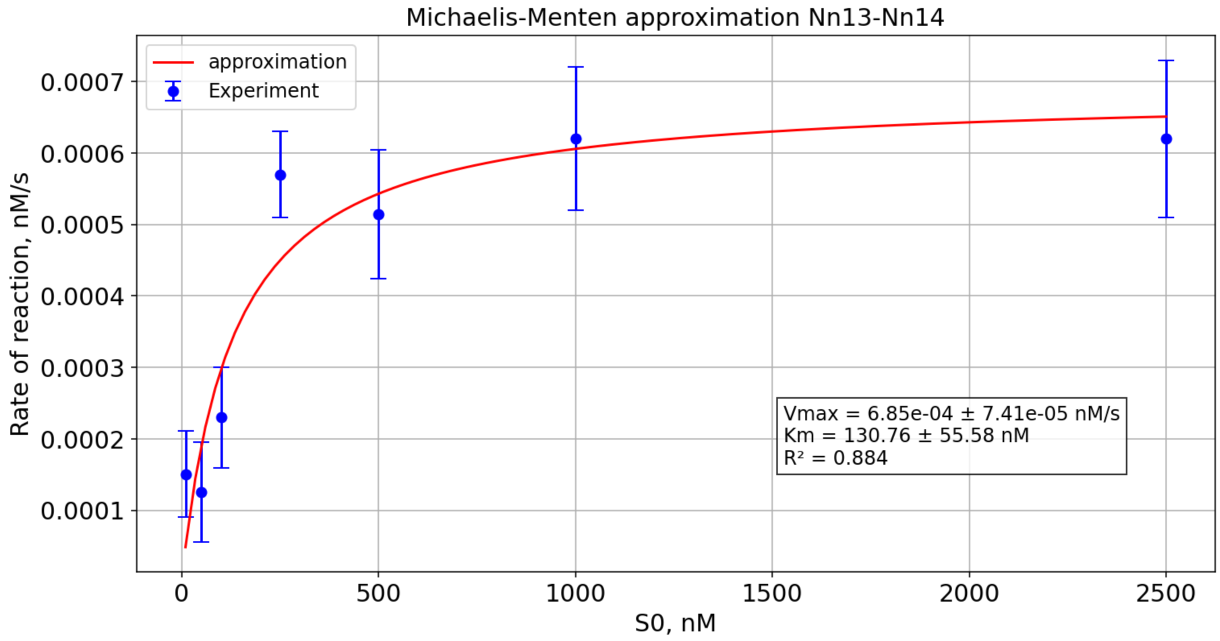

Figure S30. Approximation for duplex Nn13*-Nn14.

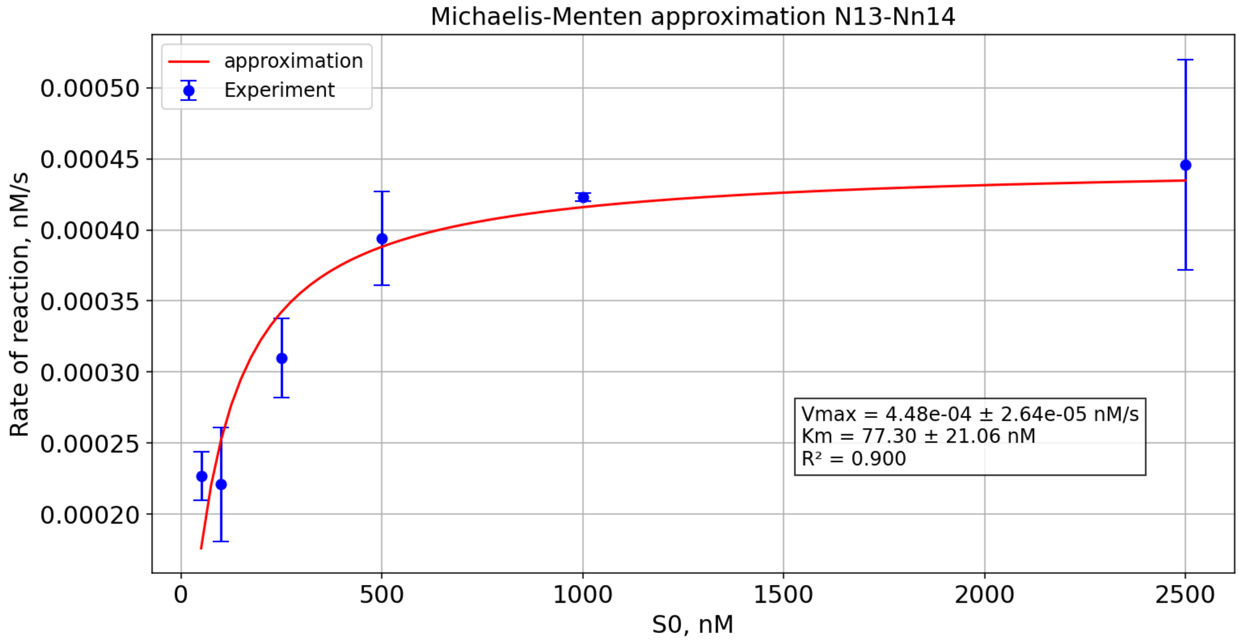

Figure S31. Approximation for duplex N13*-Nn14.

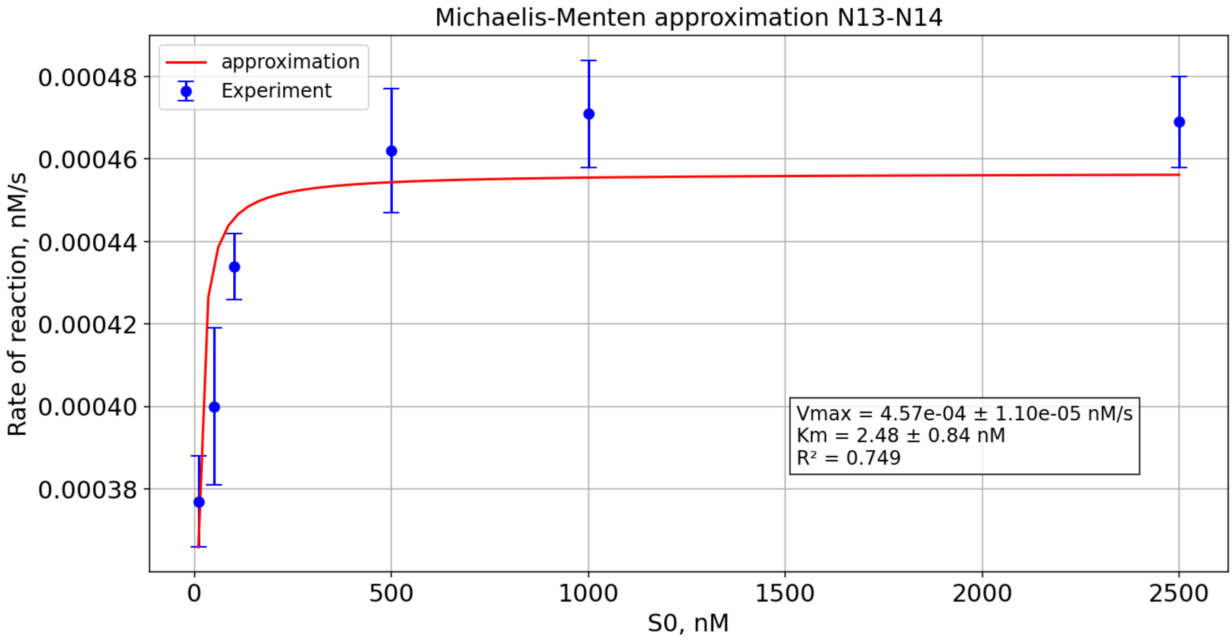

Figure S32. Approximation for duplex N13*-N14.
